# Protein Arginine Methyltransferases Refine the Classification of Clear Cell Renal Cell Carcinoma with Distinct Prognosis and Tumor Microenvironment Characteristics

**DOI:** 10.7150/ijbs.80323

**Published:** 2023-08-28

**Authors:** Shiqi Ye, Xi Tian, Aihetaimujiang Anwaier, Shiyin Wei, Wangrui Liu, Jiaqi Su, Shuxuan Zhu, Bo Dai, Jun Gu, Yuanyuan Qu, Wenhao Xu, Hailiang Zhang, Dingwei Ye

**Affiliations:** 1 Department of Urology, Fudan University Shanghai Cancer Center, Shanghai 200032, P.R. China.; 2 Department of Oncology, Shanghai Medical College, Fudan University, Shanghai 200032, P.R. China.; 3 Shanghai Genitourinary Cancer Institute, Shanghai 200032, P.R. China.; 4 Department of Neurosurgery, Affiliated Hospital of Youjiang Medical University for Nationalities, Baise, 533000, P.R. China.; 5 Department of Interventional Oncology, Renji Hospital, Shanghai Jiao Tong University School of Medicine, Shanghai, 200127, China.; 6 The Affiliated Jiangsu Shengze Hospital of Nanjing Medical University, Suzhou 215228, P.R. China.

**Keywords:** clear cell renal cell carcinoma, protein arginine methylation, prognosis, tumor microenvironment, immunotherapy response, PRMT5

## Abstract

**Background:** Clear cell renal cell carcinoma (ccRCC) is an aggressive urological cancer that originates from the proximal tubular epithelium. As one of the most common post-translational modification, protein arginine methylation plays a pivotal role in various cancer-associated biological functions, especially in cancer immunity. Therefore, constructing a protein arginine methylation-related prognostic signature would be beneficial in guiding better personalized clinical management for patients with ccRCC.

**Methods:** Based on the multi-omics profiling of the expression levels of eight protein arginine methyltransferases (PRMTs) in 763 ccRCC samples (from TCGA, CPTAC, EMBL, and ICGC databases), we established a scoring system with machine-learning algorithms to quantify the modification patterns on clinical and immunological characterizations of individual ccRCC patient, which was termed as PRMTScore. Moreover, we utilized two external clinical cohorts receiving immunotherapy (n=302) to validate the reliability of the PRMTScore system. Multiplex immunohistochemistry (mIHC) was performed to characterize the cellular composition of 30 paired ccRCC samples. The proteomic profiling of 232 ccRCC samples obtained from Fudan University Shanghai Cancer Center (FUSCC) was analyzed to validate the protein expression of PRMT5 in ccRCC. Finally, CCK-8, transwell, and wound healing assays were conducted to elucidate the role of PRMT5 in ccRCC in vitro.

**Results:** A total of 763 ccRCC patients with available multi-omics profiling were stratified into two clusters (PRMTCluster A and B) with distinctive prognosis, genomic alterations, tumor microenvironment (TME) characteristics, and fundamental biological mechanisms. Subsequently, protein arginine methylation-related prognostic signature (PRMTScore) was constructed and consisted of *SLC16A12, HRH2, F2RL3,* and *SAA1*. The PRMTScore showed remarkable differences in outcomes, immune and stromal fractions, expressions of immune checkpoints, the abundance of immune cells, and immunotherapy response in ccRCC patients. Additionally, preliminary insights unveiled the tumor-suppressive role of PRMT5 in ccRCC, and the signal of PRMT5^low^ significantly predicted aggressive prognosis and the high abundance of PD1^+^ CD8^+^ cells in ccRCC.

**Conclusion:** We constructed a PRMTScore system, which showed the potent ability to assess the prognosis, TME characteristics, and immunotherapy response for patients with ccRCC. Moreover, this is the first study to propose that PRMT5 acts as a cancer suppressor in ccRCC.

## Introduction

Renal cell carcinoma (RCC) is among the top ten most common malignant carcinomas in America in 2022 1. In China, it has been estimated that approximately 75,800 new cases and 26,900 deaths occurred in 2016 due to RCC 2. Clear cell renal cell carcinoma (ccRCC), accounting for ~80% of RCC cases, is an aggressive histological subtype that originates from the proximal tubular epithelium 3,4. In the Chinese and Caucasian populations with ccRCC, the genes with the highest frequency of somatic mutations are VHL, PBRM1, and BAP1 5. Intra-tumoral heterogeneity (ITH) due to these mutational events appears to be a typical feature of ccRCC 6. Since various targeted agents were developed into the treatment strategies of ccRCC patients, such as inhibitors of vascular endothelial growth factor (VEGF) and mammalian target of rapamycin (mTOR), the prognostic outcomes of patients have been greatly improved 7. However, the treatment response and efficacy of targeted therapies are varied, and most patients eventually progress to death 8.

In recent years, the rapid development of immune checkpoint inhibitor (ICI)-based immunotherapy has ushered in a promising era of anticancer therapy. Persistent response can be seen in patients with melanoma, non-small-cell lung cancer (NSCLC), and other malignant tumors 9,10,11. Since the U.S. Food and Drug Administration (FDA) approved nivolumab, an anti-PD-1 monoclonal antibody, for the treatment of advanced ccRCC (accRCC) patients in 2015, novel immunotherapies and immunotherapy-based combinatorial strategies have revolutionized the treatment paradigm for patients with ccRCC 12,13. Despite these advances, only a small proportion of ccRCC patients obtain durable benefits from ICI agents, mainly attributed to the complex individual heterogeneity 14,15. Therefore, identifying the TME characteristics in-depth and elucidating accurate predictive models are significant in developing better personalized ICI treatment strategies and optimizing clinical outcomes for patients with ccRCC 16.

Post-translational modifications (PTMs) change the biophysical properties of proteins, which results in the diversity of their stability, interactions, and functions. As one of the most common types of PTMs, protein arginine methylation is a process in which protein arginine methyltransferases (PRMTs) catalyze the transfer of a methyl group from S-adenosylmethionine (SAM) to the guanidino nitrogen atoms of arginine 17. The methylarginine products include: ω‐*N*^G^‐monomethylarginine (MMA), ω‐*N*^G^,*N*^G^‐asymmetric dimethylarginine (aDMA) and ω‐*N*^G^,*N*'^G^‐symmetric dimethylarginine (sDMA) 18. So far, a total of 9 PRMTs have been identified that can be catagorized into three types. Type I PRMT includes PRMT1, PRMT2, PRMT3, CARM1 (also known as PRMT4), PRMT6, and PRMT8, and the members of this type catalyze the formation of aDMA. Type II PRMT (PRMT5 and PRMT9) catalyzes the formation of sDMA. Type III PRMT (PRMT7) only catalyzes the formation of MMA 19. In comparison to the PRMTs (“writers”), the existence of specific arginine demethylases (“erasers”) remains controversial, and further investigations are required to identify the enzymes necessary to reverse methylated arginine 20,21. Protein arginine methylation is closely involved in the regulation of a wide range of fundamental cellular processes, including transcription, splicing, DNA damage response, and cell metabolism, which are linked to numerous diseases 19,22. In cancer research, protein arginine methylation is also well investigated. Multiple PRMT substrates play critical roles in regulating cancer-associated epigenetics, transcription, signaling, RNA metabolism, and DNA repair 18,23. More importantly, protein arginine methylation has been reported to function as the regulator of cancer immunity 24. Some researches revealed that the knockout or inhibition of PRMTs, in combination with ICI agents, played a synergistic effect in the treatment of melanoma, colon adenocarcinoma, and pancreatic cancer 25,26,27,28. These findings established a rationale for the immuno-oncology therapy strategies based on PRMTs. To date, several studies have investigated the roles of PRMTs (PRMT1, CADM1, and PRMT7) in ccRCC 29,30,31. However, the impact of PRMTs on TME characteristics and clinical outcomes of ccRCC patients is still uncertain 32.

In this study, we systematically analyzed the underlying effects of PRMTs in ccRCC. Moreover, a protein arginine methylation-related signature was constructed to predict the prognostic outcomes, TME characteristics, and immunotherapy response in ccRCC patients. Our findings uncovered an important role of protein arginine methylation modification in complex heterogeneity of ccRCC, which may contribute to improvement in the individualized management of ccRCC patients. Besides, this was the first study to propose the potential anticancer function of PRMT5 in ccRCC, which would provide a foundation for the in-depth exploration of the promising molecular in ccRCC.

## Materials and Methods

### Data Collection and Normalization

The transcriptome data and clinical information of 763 ccRCC patients were retrieved from the Cancer Genome Atlas (TCGA) database (https://portal.gdc.cancer.gov/repository), clinical proteomic tumor analysis consortium (CPTAC) database (https://cptac-data-portal.georgetown.edu/studysummary/S050), European Molecular Biology Laboratory (EMBL) database (https://www.ebi.ac.uk/arrayexpress/), and International Cancer Genome Consortium (ICGC) database (https://icgc.org/), including TCGA-KIRC, CPTAC-3, E-MTAB-3267, and RECA-EU datasets or projects. The expression levels of the RNA-seq samples were converted from fragments per kilobase of transcript per million mapped reads (FPKM) to transcripts per million (TPM), and log2(TPM + 1) was taken. The four sets of expression data were combined and we intersected the same genes in different cohorts for further analyses. Besides, we eliminated the batch effect based on the 'ComBat' algorithm of sva package. Somatic mutation data, copy number variations (CNVs) files, and tumor mutation burden (TMB) data of ccRCC patients were obtained from the TCGA database. The detailed baseline clinical data of patients was summarized in **Table S1**.

### Consensus Clustering Analysis

Consensus clustering analysis was applied to stratify ccRCC patients into different subgroups. We conducted the “ConsensusClusterPlus” package in R to identify the optimal number of clusters and the distribution of patients, and 1000 iterations were performed to ensure the stability of the results 33.

### Association between the Molecular Patterns and Clinical Characteristics

The clinical *factors* included age, gender, tumor grade, and the TNM stage. Moreover, the difference in prognostic outcomes among distinct patterns was evaluated with Kaplan-Meier analysis using the “survival” and “survminer” packages in R software 34.

### Exploration of the Immune Landscape in Distinct Molecular Patterns

The abundance of 22 immune cell subtypes in 763 ccRCC specimens were assessed with the CIBERSORT algorithm in R software 35. The infiltrating fractions of immune cells were also identified with the single-sample gene set enrichment analysis (ssGSEA) algorithm 36. Subsequently, we evaluated the immune score, stromal score, ESTIMATE score, and the tumor purity of each ccRCC samples with the ESTIMATE algorithm 37. Moreover, we compared the expression levels of immune checkpoints (ICPs) among the different patient subgroups.

### Differential Expression and Functional Enrichment Analyses

Differential expression genes (DEGs) between different arginine methylation modification patterns were identified using the “limma” package in R software, with the screening criteria of |log2-fold change (FC)| > 1 and adjusted P value < 0.001 38. Based on the DEGs, Gene Ontology (GO) annotation and Kyoto Encyclopedia of Genes and Genomes (KEGG) pathway enrichment analyses were performed with the “clusterProfiler” package to compare different biological functions and signal pathways 39. Furthermore, we conducted the gene set variation analysis (GSVA) with the KEGG gene set (c2.cp.kegg.v7.5.1), to identify the biological functional differences in different subgroups, with the criteria of |log2-fold change (FC)| > 0.2 and adjusted P value < 0.001 40.

### Construction of PRMTs-Related Predictive Signature

A prognostic scoring system (PRMTScore) was established in this study. First, we performed the univariate Cox regression analysis with the expressions of DEGs and survival information to determine the prognosis-related genes. Subsequently, the least absolute shrinkage and selector operation (LASSO) and multivariate Cox analysis were conducted to create an optimal predictive model. The PRMTScore was assessed with the selected genes as described: PRMTScore = h_0_(t) * exp (expression of SLC16A12 * corresponding coefficient + expression of HRH2 * corresponding coefficient + expression of F2RL3 * corresponding coefficient + expression of SAA1 * corresponding coefficient). Patients were stratified into low and high PRMTGroups, utilizing the median PRMTScore as the threshold.

### Evaluation of the Clinical Significance of the Predictive Signature

The survival difference between the two PRMTGroups was analyzed through the Kaplan-Meier analysis. The receiver operating characteristic (ROC) curve was constructed to validate the predictive ability of the PRMTScore system using the 'survival ROC' R package. Additionally, the signature's prognostic potential was explored through the stratification of ccRCC patients based on clinical characteristics.

### Establishment of a Nomogram for Predicting Prognostic Outcomes

Based on the PRMTScore and clinical characteristics, we constructed a nomogram to predict 1-, 3-, and 5-year overall survival (OS) for ccRCC patients using the “rms”, “regplot”, and “survival” packages in R software. Next, calibration curves, time-dependent ROC curves, and decision curve analysis (DCA) were performed to verify the accuracy and stability of the nomogram.

### Prediction of the Treatment Response to ICI Therapy

We applied tumor immune dysfunction and exclusion (TIDE) analysis to predict the ICI treatment response of ccRCC patients. The analytic technique TIDE enables the prediction of immunotherapy response with two major tumor immune evasion mechanisms: T cell dysfunction and T cell infiltration inhibited in tumor with low cytotoxic T lymphocyte (CTL) levels 41. Patients with low TIDE score are predicted to respond to immunotherapy. In addition, we used two ICI treatment cohorts to validate the immunotherapy response of the PRMTs-related signature. We obtained the David A. Braun cohort from a previously reported study, which included 181 nivolumab (anti-PD-1 blockage) treated accRCC patients 42. The David Liu cohort consisted of 121 metastatic melanoma patients who were treated with nivolumab or pembrolizumab (anti-PD-1 blockages) 43.

### External Validation of PRMT5 Protein Expression

First, we used the immunohistochemistry (IHC) staining slides from the Human Protein Altas (HPA) (https://www.proteinatlas.org). Next, to characterize the cellular compositions of ccRCC and adjacent tissues, we conducted multiplex immunohistochemistry (mIHC) in 30 paired ccRCC and adjacent tissues. The tissue microarrays were obtained from Shanghai Wellbio Biotechnology Co., Ltd (Wellbio Biotechnology Co.,Shanghai, China). We investigated the abundance of PRMT5 (A19533; ABclonal), CD8 (ab217344; Abcam), and PD1 (ab52587; Abcam) in 30 ccRCC and adjacent tissues. The CaseViewer software was applied to visualize and obtain the images. Moreover, the proteomic profiling of 232 ccRCC and paired adjacent samples obtained from Fudan University Shanghai Cancer Center (FUSCC) were analyzed to validate the expression of PRMT5 in ccRCC. All patients provided consent for the examination and signed an informed consent form. The Helsinki Declaration II was followed for the design of the study and the testing techniques. Further, the Fudan University Shanghai Cancer Center's ethical committee approved the study methods utilized in this research (FUSCC, Shanghai, China).

### Cell Culture and Reagents

Human ccRCC 786-O and 769-P cells were purchased from the Type Culture Collection Cell Bank, Chinese Academy of Sciences. The cells were grown in RPMI 1640 medium supplemented with 10% fetal bovine serum and 1% penicillin/streptomycin. The cells were cultured at 37 °C with 5% CO2.

### Cell Transfection

The cells were planted in a 10-cm dish for 50% density a day before transfection. Transfection could be performed when the cells grew to a density of 70%. The negative control and PRMT5 overexpression plasmids were respectively mixed with Lipofectamine 3000 (Invitrogen, Carlsbad, CA, USA) based on the manufacture's guide. After incubation for 15 min at room temperature, we added the mixtures to the cell culture dish. Cells were harvested 48 hours after transfection for further analysis.

### Western Blotting

Cells were harvested by scraping into an SDS sample buffer containing a cocktail of protease inhibitors and PhosSTOP Phosphatase Inhibitor (Roche, Pleasanton, CA, USA). Western blotting was conducted according to the standard procedure. We investigated the abundance of PRMT5 (A19533; ABclonal) and β-Actin (ab8226; Abcam).

### Cell Counting Kit (CCK)-8 Assay

We first seeded cells into 96-well plates (5000 cells/well) with 100 µl complete culture medium. After incubation for 1, 2, 3, and 4 days, respectively, 10 µl CCK-8 solution was added to each well. Subsequently, the cells were cultured for an additional 2 hours while ensuring that light was avoided. The OD values of the cells were detected using a Microplate Spectrophotometer (BioTek, VT, USA) at 450 nm wave length.

### Transwell Assay

A total of 20,000 cells were seeded in the top of a polycarbonate Transwell filter with 200 µl culture medium (without fetal bovine serum). The lower compartment was filled with 800 µl complete culture medium. A layer of Matrigel was spread on the upper surface of the Transwell filter. After incubation for 24 hours, the cells were fixed with 4% paraformaldehyde solution and stained with crystal violet.

### Wound Healing Assay

We planted 500,000 cells into 6-well plates. When the cells were overgrown, we scratched the cells using 100-µl pipette tips to create a wound. After 24 hours, we evaluated the cell migration capacity by measuring the wound gap area.

### Statistical Analysis

All statistical analyses and graph visualizations were conducted using R v4.1.3 and GraphPad Prism v9.4.0. The Wilcoxon rank-sum test was applied to compare the differences between the two groups. The Kaplan-Meier method was utilized to perform survival analysis, and log-rank test was used to determine the significance. A *P*-value of less than 0.05 was considered statistically significant unless stated otherwise.

## Results

### The Landscape of Expression Levels and Genomic Variations of PRMTs in ccRCC

**Figure 1:**
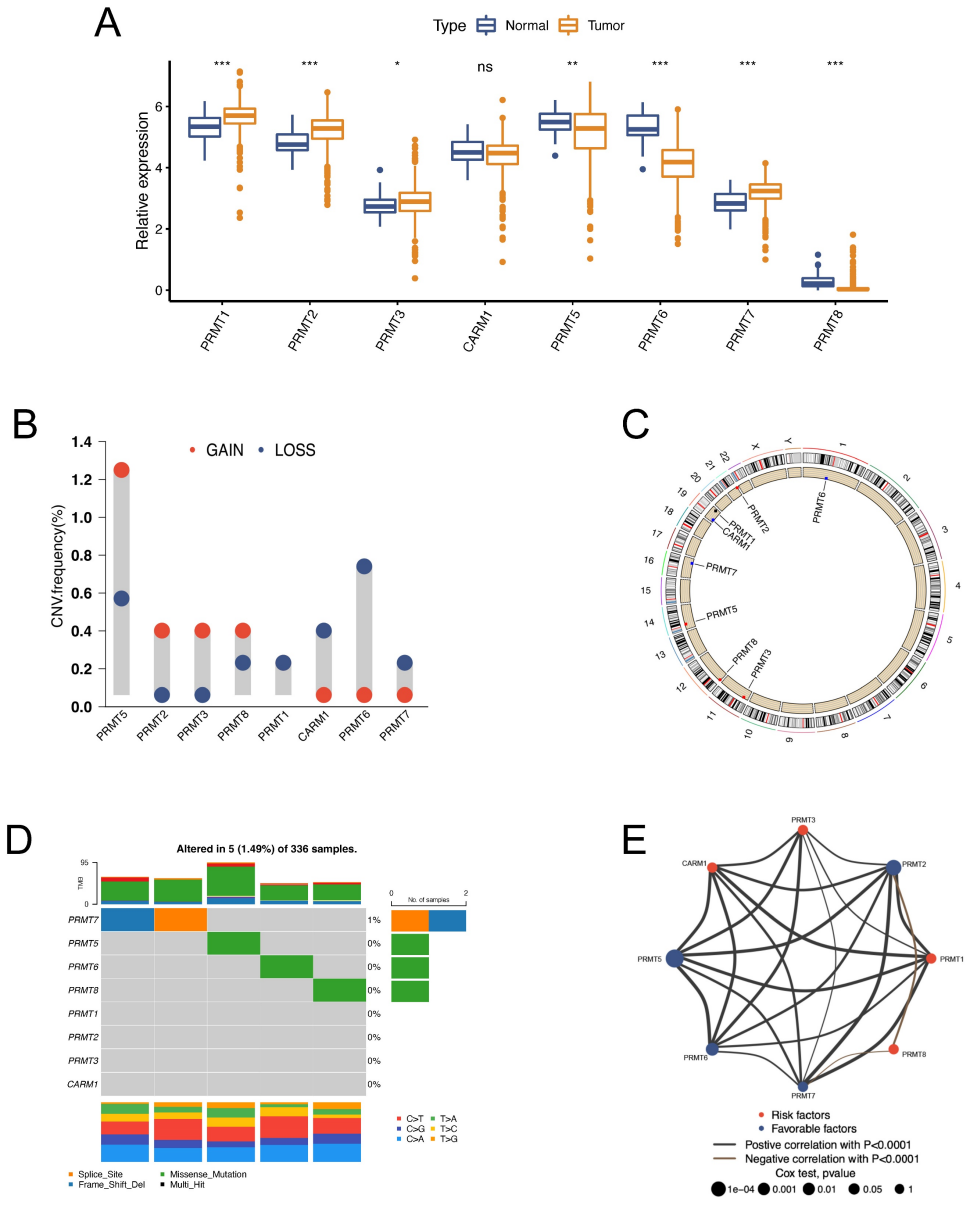
** The landscape of expression levels and genomic alterations of the 8 protein arginine methyltransferases (PRMTs) in clear cell renal cell carcinoma (ccRCC) patients.** (A) Expressions of the 8 PRMTs between tumor and normal specimens in the TCGA-KIRC cohort. (B) Alterations of copy number variations (CNVs) frequency of the 8 PRMTs. (C) Locations of alterations in CNVs of the 8 PRMTs on 23 chromosomes. (D) Mutation frequency and types of the 8 PRMTs. (E) The network of correlations and prognostic significances of the 8 PRMTs in ccRCC patients from the TCGA-KIRC, CPTAC-3, E-MTAB-3267, and RECA-EU cohorts. (ns, not significant; *p < 0.05, **p < 0.01, ***p < 0.001).

All the recognized PRMTs, except PRMT9, were analyzed in this study **Table S2**. The mRNA expressions of the 8 PRMTs in tumor and normal specimens were assessed using the TCGA-KIRC dataset. Notably, PRMT1, PRMT2, PRMT3, and PRMT7 exhibited upregulation, while PRMT5, PRMT6, and PRMT8 displayed downregulation in tumor (Figure 1A). Further examination was conducted to evaluate copy number variations (CNVs) across the eight PRMTs in ccRCC. The results, illustrated in **Figure 1B**, revealed a low frequency of CNVs among these PRMTs. Specifically, PRMT5, PRMT2, PRMT3, and PRMT8 displayed amplification in copy numbers, whereas CARM1, PRMT6, and PRMT7 exhibited copy number deletions. The locations of these CNV alterations on chromosomes were visualized in **Figure 1C**. Furthermore, the incidence of somatic mutations across the eight PRMTs in ccRCC was assessed, with only 5 out of 336 ccRCC specimens (1.49%) displaying genetic mutations (**Figure 1D**). The above findings revealed that there were relatively infrequent CNVs alterations and somatic mutations of PRMTs in ccRCC. The prognostic value of the 8 PRMTs in ccRCC patients was identified with uniCox and Kaplan-Meier analysis Figure S1 and Table S3. Subsequently, a comprehensive regulatory network was constructed to illustrate the comprehensive landscapes of the correlations and prognostic significances of the eight PRMTs in ccRCC patients. Notably, PRMT2, PRMT5, and PRMT6 exhibited significant correlations with an adverse prognosis (Figure 1E and Table S4).

### Generation of Arginine Methylation Modification Patterns and Identification of the Differences in the Subgroups

**Figure 2:**
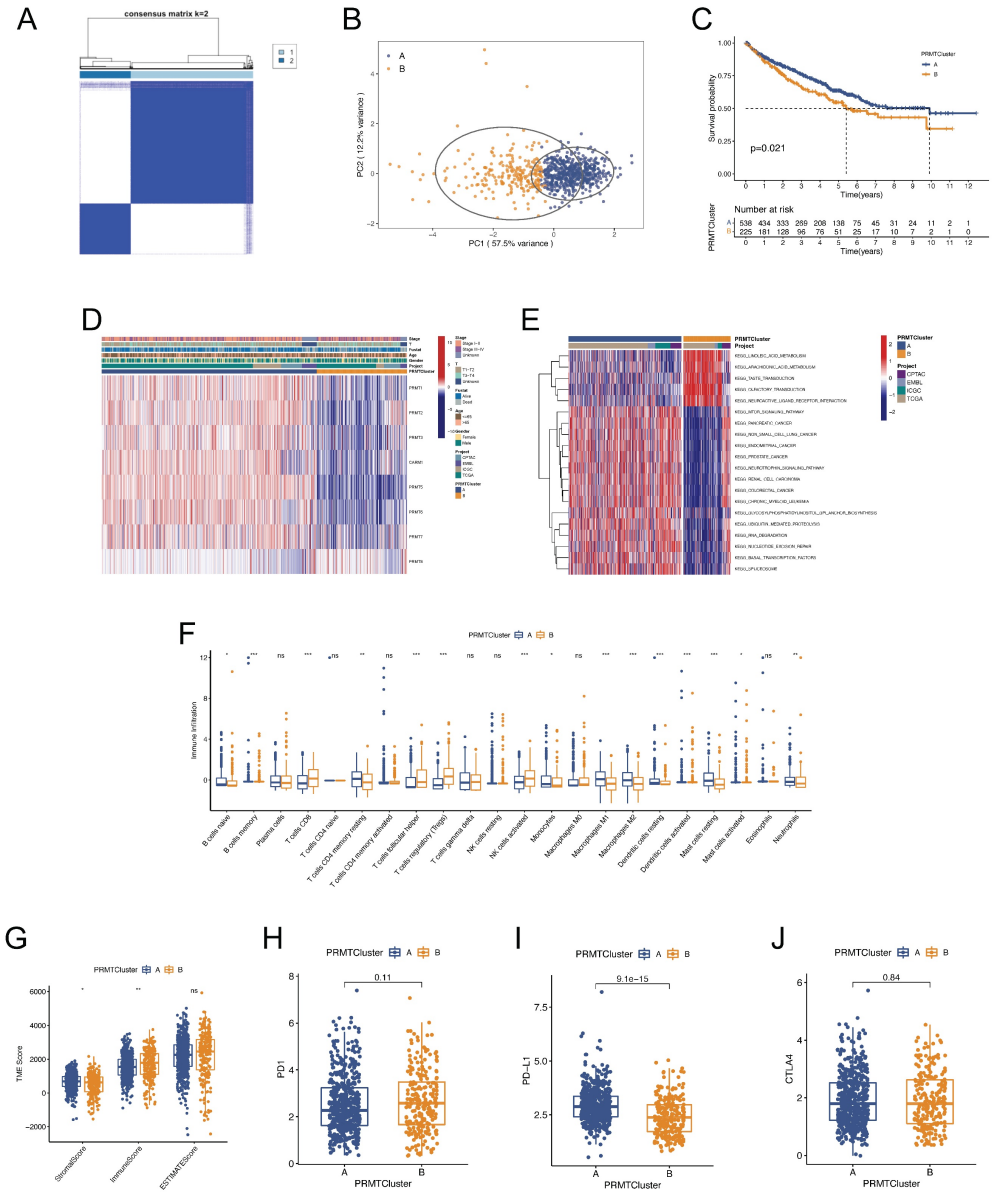
** Clinical and biological characteristics of different PRMTClusters.** (A) Consensus matrix heatmap defining the two PRMTClusters and their correlation area. (B) Principal component analysis (PCA) indicating the differences in transcriptomes between the two subgroups. (C) Kaplan-Meier curves of the overall survival (OS) for patients in different PRMTClusters. (D) Differences in the clinical characteristics and expressions of the 8 PRMTs between PRMTClusters. (E) Gene set variation analysis (GSVA) demonstrating activation states of biological pathways between PRMTClusters. (F) Abundance of 22 infiltrating immune cell types in PRMTClusters. (G) Differences in the tumor microenvironment (TME) scores between PRMTClusters. (H-J) Expressions of PD-1, PD-L1, and CTLA-4 in PRMTClusters. (ns, not significant; *p < 0.05, **p < 0.01, ***p < 0.001).

To further recognize the effects of PRMTs in ccRCC, consensus clustering analysis was undertaken to stratify ccRCC patients into different arginine methylation modification patterns, termed as PRMTClusters. The optimal number of the clusters was 2, and more patients were distributed in PRMTCluster A (538) than that in PRMTCluster B (225) (Figure 2A and Table S5). The results were defined by the least crossover in the consensus matrixes, the smooth trend in the cumulative distribution function (CDF) curves, and no significant shift in the area under the CDF curves Figure S2. Based on the expression profiles of the 8 PRMTs, the principal component analysis (PCA) confirmed an excellent intergroup distribution (Figure 2B). Furthermore, we compared the OS of ccRCC patients between the two PRMTClusters, indicating that patients in PRMTCluster A had a favorable prognosis (Figure 2C). The expressions of PRMTs and clinical characteristics in the two clusters were illustrated in a heatmap (Figure 2D). Besides, the GSVA analysis uncovered activation of cancer-associated pathways in PRMTcluster A (Figure 2E and Table S6). Notably, it has been reported that the mTOR signaling pathway was frequently activated in human cancer 44. To identify the impacts of PRMTs on the TME characteristics of ccRCC, we explored the infiltrating levels of 22 human immune cell types in the two clusters with the CIBERSORT algorithm Table S7. As demonstrated in Figure 2F, most immune cells showed great enrichment differences. Furthermore, to evaluate the abundance of immune and stromal fractions in TME, we performed the ESTIMATE algorithm to assess the TME scores in the PRMTClusters, which included the stromal, immune, and estimate scores. The result indicated that there were more immune cells and fewer stromal cells fractions in PRMTCluster A (Figure 2G and Table S8). Moreover, the expression differences of crucial immune checkpoints (ICPs) including PD-1, PD-L1, and CTLA-4 were explored, revealing a notable elevation of PD-L1 in PRMTCluster A (Figures 2H-J). Taken together, we identified two PRMTClusters based on the expression profiling of PRMTs, and analyzed the differences in prognosis, biological functions, and TME characteristics of patients between the two PRMTClusters.

### Functional Annotations and Identification of Genetic clusters Based on the DEGs

**Figure 3:**
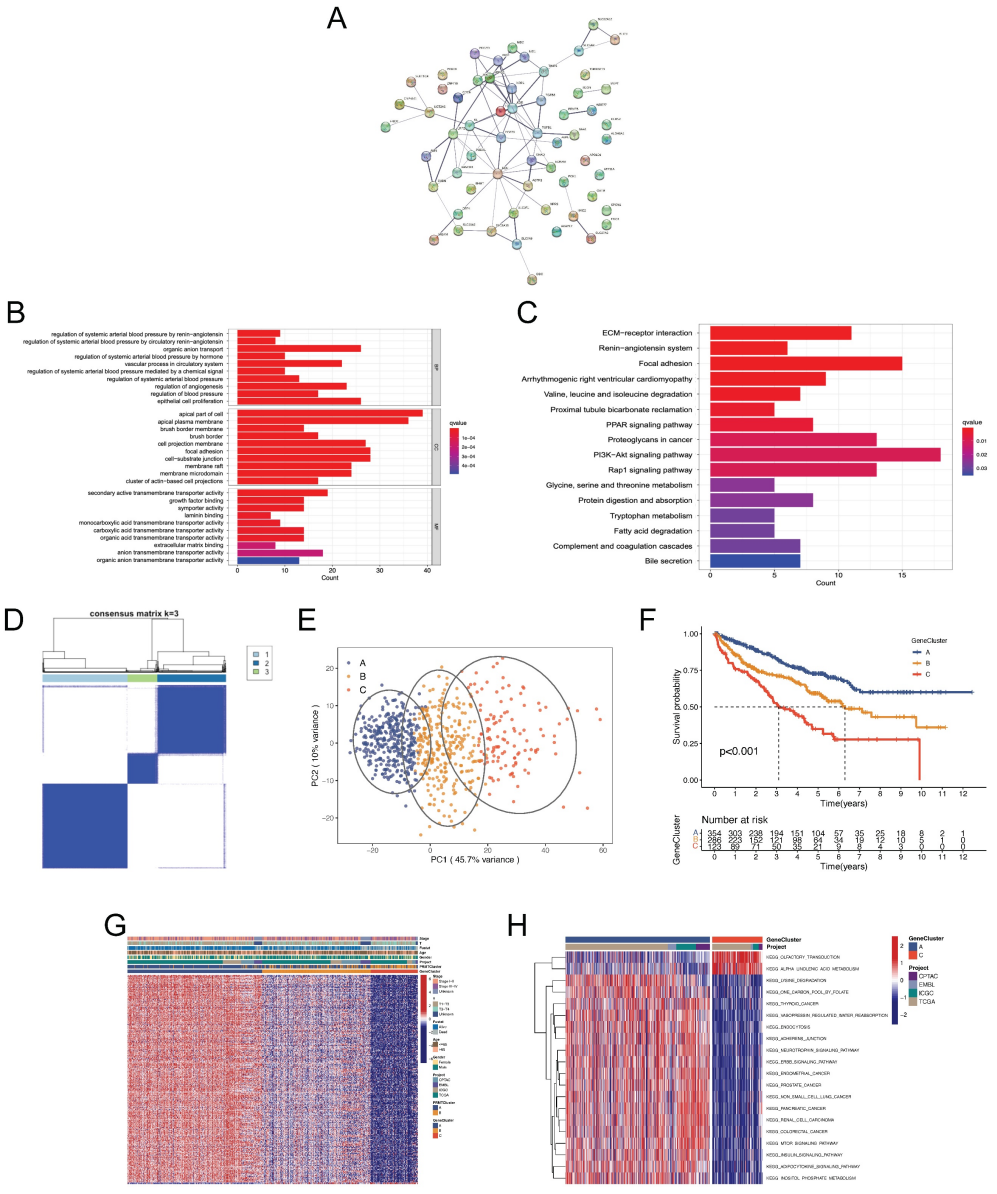
** The functional annotation and identification of genetic subgroups based on the differential expression genes (DEGs).** (A) The protein protein interaction (PPI) network showing the interactions among the top 50 DEGs between the two PRMTClusters. (B, C) Gene ontology (GO) and kyoto encyclopedia of genes and genomes (KEGG) enrichment analysis of DEGs. (D) The consensus matrix heatmap defining different genetic clusters based on the 294 prognosis-related genes. (E) PCA for the transcriptome profiles of the three GeneClusters. (F) Kaplan-Meier curves for OS of patients in different GeneClusters. (G) Relationships between clinical features and GeneClusters. (H) GSVA showing the activation states of biological pathways between GeneClusterA and B.

To further recognize the differences in biological behaviors of ccRCC between the PRMTClusters, we identified 312 PRMTCluster-associated DEGs using the “limma” package Table S9. A protein protein interaction (PPI) network among the top 50 DEGs was constructed from the STRING database (Figure 3A).

Subsequently, functional enrichment analyses were performed, yielding significant insights into biological processes, molecular functions, and cellular components (Figure 3B and Table S10). KEGG analysis highlighted the top 3 significantly abundant pathways in cancer: PI3K-Akt signaling pathway, focal adhesion, and proteoglycans (Figure 3C and Table S10), indicative of the pivotal role of protein arginine methylation in regulating ccRCC growth, progression, and metastasis. UniCox analysis was employed to assess the survival significance of DEGs, leading to the identification of 294 genes with prognostic relevance with the screening criterion of p < 0.05 Table S11. The consensus clustering algorithm was employed to stratify patients into different genetic clusters, termed as GeneCluster, based on the 294 prognosis-related genes (Figure 3D and Table S5). The CDF, delta, and tracking plots corresponding to the consensus matrixes were displayed in Figure S3. These GeneClusters exhibited distinct transcriptome profiling, as evidenced by PCA (Figure 3E). Subsequent Kaplan-Meier analysis demonstrated that patients in GeneCluster A had the longest OS time, whereas patients in GeneCluster C suffered the worst prognosis (Figure 3F). Besides, a heatmap was generated to elucidate the differences in clinical characteristics across the three GeneClusters (Figure 3G). Notably, patients in GeneCluster C had a more advanced TNM stage, consistent with the result of survival analysis. GSVA enrichment analysis unveiled varying activation states of biological pathways among these distinct subgroups (Figure 3H, Figure S3 and Table S12). Finally, we analyzed the tumor immune microenvironment characteristics of different GeneClustes Figure S3. Interestingly, GeneCluster C had the highest infiltration level of T cells regulatory (Tregs), suggestive of immunosuppression and congruent with the worsened prognosis observed (Figure 3F).

### Construction of a PRMTs-related Predictive Signature

**Figure 4:**
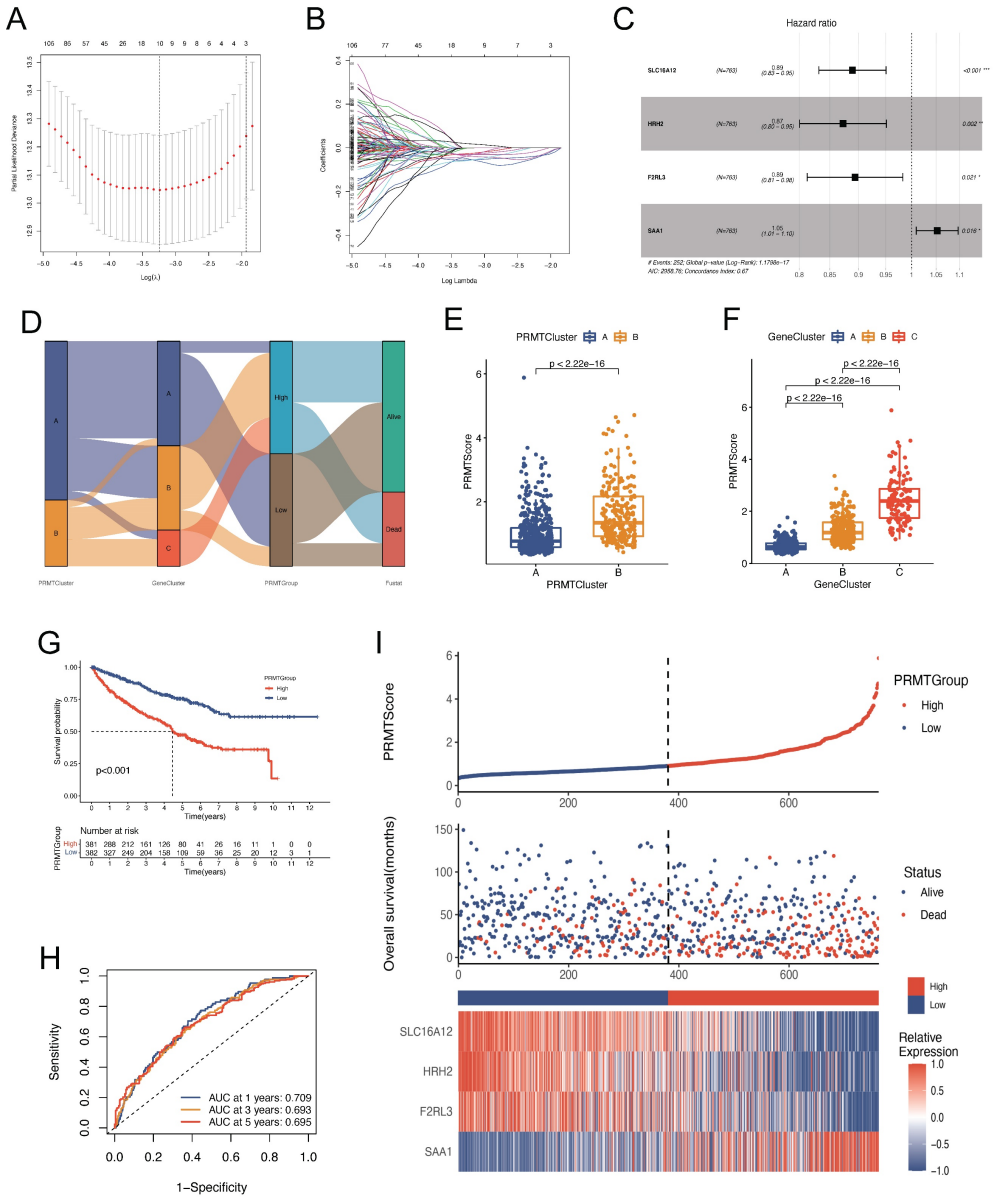
** Construction of a PRMTs-related predictive signature.** (A-B) Coefficient profiles of 294 prognosis-related genes and identification of the best parameter (lambda) according to the least absolute shrinkage and selector operation (LASSO). (C) Four genes identified to construct the optimal PRMTs-related predictive signature using multivariate Cox analysis. (D) The alluvial diagram showing the correlations among PRMTClusters, GeneClusters, PRMTScore, and clinical outcomes. (E) Differences in patients' PRMTScore between the two PRMTClusters. (F) Differences in patients' PRMTScore among the three GeneClusters. (G) Kaplan-Meier analysis of OS for patients in different PRMTGroups. (H) The receiver operating characteristic (ROC) curves to predict the sensitivity and specificity of 1-, 3-, and 5-year OS according to the PRMTScore. (I) The scatter plot of distributions of the PRMTScore and survival status, and the heatmap of expressions of the four selected genes.

The preceding outcomes underscore the pivotal role of arginine methylation modification in shaping the prognostic outcomes and tumor microenvironment (TME) characteristics of ccRCC patients. Nevertheless, these analyses, while informative for the patient population, do not facilitate accurate prognostication of individual patient. Considering the individual heterogeneity and complexity of arginine methylation modification, we constructed a PRMTs-related signature to predict the prognosis of ccRCC patients. We established the scoring system, termed as PRMTScore, based on the 294 prognosis-associated DEGs. LASSO and multivariate Cox analyses were conducted to create an optimal predictive model (Figure 4A-C). We identified four genes that had the greatest prognostic significance for constructing the predictive tool, including *SLC16A12*, *HRH2*, *F2RL3*, and *SAA1* **Table S13**. Notably, *SAA1* implied a poor prognosis, while the other three genes exhibited associations with favorable outcomes. Figure 4D displayed the distribution of patients in PRMTClusters, GeneClusters, and PRMTGroups. Notably, patients in PRMTCluster B and GeneCluster C had higher PRMTScore (Figure 4E-F and Table S5). Furthermore, Kaplan-Meier analysis confirmed that patients characterized by a high PRMTScore experienced worse OS, a trend congruent with those observed within PRMTCluster B and GeneCluster C (Figure 4G, 2C,** and** 3F). The area under the curves (AUCs) of the ROC curves of 1-, 3-, and 5-years OS were 0.709, 0.693, and 0.695, respectively, indicating exceptional sensitivity and specificity of the scoring system (Figure 4H). The distribution plot of the PRMTScore and survival status of patients revealed an upward trend in mortality rates with increasing PRMTScore (Figure 4I).

### Correlation between Clinical Characteristics and the PRMTs-related Predictive Signature

**Figure 5:**
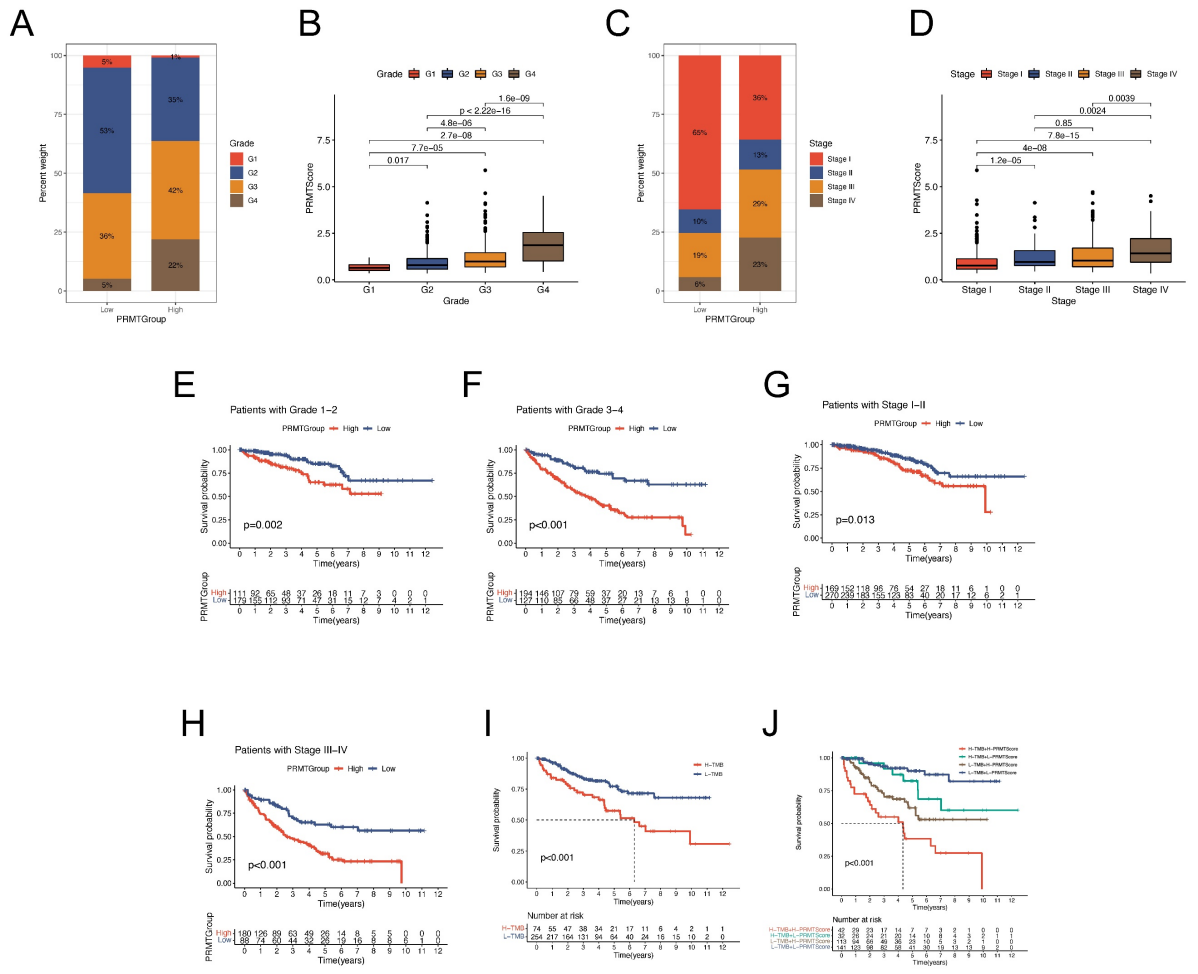
** Association between clinical information and the predictive signature.** (A-D) The proportion of clinical characteristics (tumor grade and TNM stage) of ccRCC patients in high and low PRMTGroups. (E-H) Kaplan-Meier analyses for OS of patients in different PRMTGroups stratified by clinical features (tumor grade and TNM stage). (I) Survival analysis for patients in high and low tumor mutation burden (TMB) groups. (J) Survival analysis for patients subgroups stratified by TMB and the PRMTScore.

To comprehensively validate the predictive reliability of the PRMTScore, Kaplan-Meier analyses were conducted across subgroups stratified by distinct clinical factors. We found that patients in low PRMTGroup had low tumor grade and TNM stage (Figure 5A and C). Conversely, an elevated PRMTScore was associated with more advanced tumor grade and TNM stage among patients (Figure 5B and D). Stratified survival analyses indicated that the differences in prognostic outcomes of ccRCC patients between the high and low PRMTGroups were more significant with advanced tumor grade and TNM stage (Figure 5E-H). Importantly, it was observed that patients endowed with high PRMTScore exhibited unfavorable clinical outcomes across all subgroups delineated by tumor grade, TNM stage, T stage, age, and gender (Figure 5E-H and S4G-L). Moreover, the influence of tumor mutation burden (TMB) on the prognosis of ccRCC patients was explored via survival analyses across different TMB subgroups, where patients with low TMB displayed a more favorable prognosis (Figure 5I). Subsequently, a combined analysis of TMB and PRMTScore was conducted, revealing that the prognostic advantage observed in the low-TMB group was mitigated by a high PRMTScore (Figure 5J). Collectively, the results outlined above underscore the precision and stability of the PRMTs-related signature in predicting the clinical prognosis of ccRCC patients (Figure 4-5).

### Establishment of A Nomogram for Prognosis Prediction

**Figure 6:**
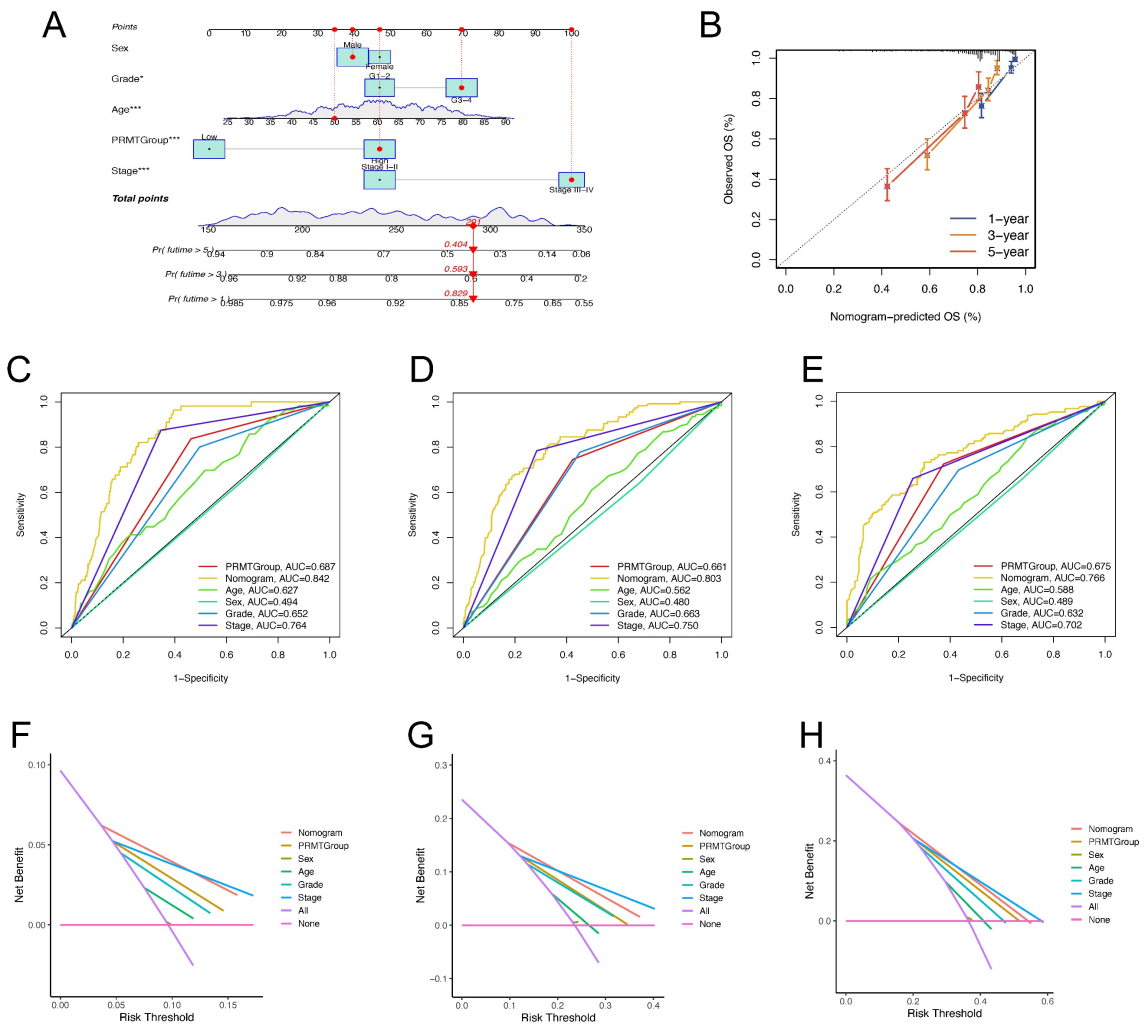
** Construction and validation of a nomogram predicting the prognosis of ccRCC patients.** (A) The nomogram predicting the 1-, 3- and 5-year OS of ccRCC patients. (B) Calibration curves for validation of the nomogram. (C-E) The time-dependent ROC curves of the nomogram for predicting the 1-, 3-, and 5-year OS. (F-H) The decision curve analysis (DCA) of the nomogram for predicting the 1-, 3-, and 5-year OS.

**Figure 7:**
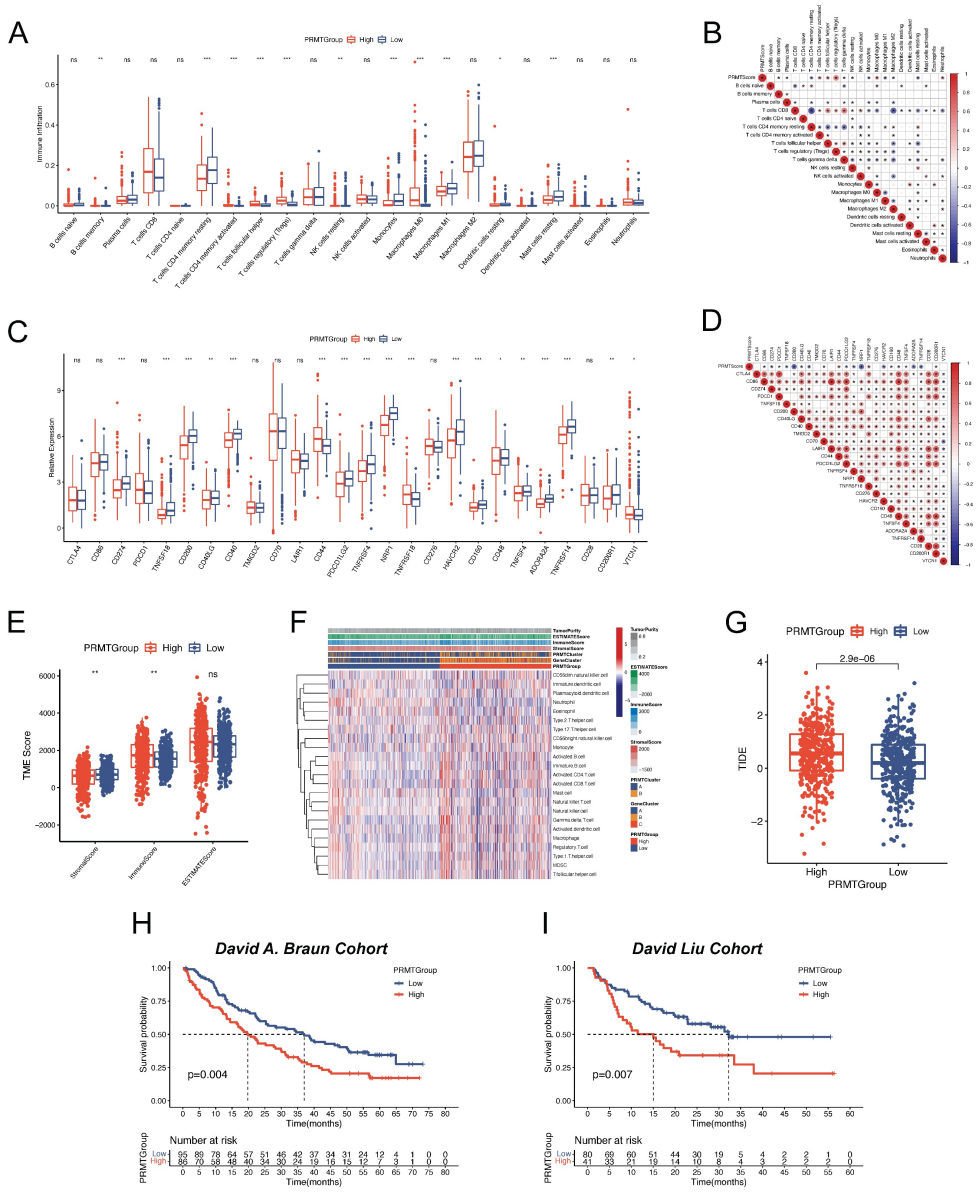
** Evaluation of TME and immunotherapy response of the predictive signature.** (A) Abundance of immune cell types in different PRMTGroups. (B) Correlation between the PRMTScore and abundance of immune cell types. (C) Expressions of immune checkpoints (ICPs) in different PRMTGroups. (D) Correlation between the PRMTScore and ICPs genes expressions. (E) Differences in the immune, stromal, and ESTIMATE scores between the two PRMTGroups. (F) The heatmap showing the distributions of TME score, PRMTClusters, GeneClusters, PRMTGroups, and the abundance of immune cell types between the two PRMTGroups. (G) Tumor immune dysfunction and exclusion (TIDE) scores in different PRMTGroups. (H) Survival analysis for patients with different PRMTScore in the David A. Braun cohort. (I) Survival analysis for patients with different PRMTScore in the David Liu cohort. (ns, not significant; *p < 0.05, **p < 0.01, ***p < 0.001).

Considering the compelling correlation between the PRMTScore system and the prognosis of ccRCC patients, we incorporated clinical characteristics with the PRMTScore to establish a nomogram for predicting the 1-, 3- and 5-year OS (Figure 6A). The calibration plot demonstrated remarkable alignment between the nomogram-predicted OS and actual OS of ccRCC patients at 1-, 3- and 5-year (Figure 7B). The AUCs of ROC curves for 1-, 3-, and 5-year OS were 0.842, 0.803, and 0.766, respectively, implying a potent predictive ability (Figure 6C-E). Besides, DCA substantiated the favorable net benefit conferred by the nomogram (Figure 6F-H). These results indicated that the nomogram had a robust power to predict the prognosis of ccRCC patients and may benefit the personalized clinical management.

### Exploration of Immune Characteristics of the PRMTs-related Predictive Signature

Since the high heterogeneity of TME affected the efficacy of immunotherapy, we further explored the immune landscape of ccRCC patients in different PRMTGroups. We first assessed the abundance of 22 immune cell types in ccRCC patients with high and low PRMTScore. Noteworthy observations emerged, revealing significantly heightened infiltrations of B cells memory, activated T cells CD4 memory, T cells follicular helper, regulatory T cells (Tregs), and macrophages M0 within the high PRMTGroup. In contrast, diminished infiltrations were discerned for T cells CD4 memory resting, resting NK cells, monocytes, macrophages M1, resting dendritic cells, and resting mast cells (Figure 7A). A correlative analysis between the abundance of immune cell types and PRMTScore was further depicted in Figure 7B. Interestingly, there was a close positive correlation of PRMTScore with the infiltration of Tregs, and a negative correlation with the infiltration of macrophages M1. Considering the importance of ICI in the clinical treatment of ccRCC patients, we analyzed the differences in the expression levels of ICPs between the PRMTGroups. This analysis uncovered significant differences in the expression levels of CD274, TNFSF18, CD200, CD40, CD44, PDCD1LG2, TNFSF4, NRP1, TNFRSF18, HAVCR2, CD160, ADORA2A, and TNFRSF14 between the two PRMTGroups (Figure 7C). Specifically, CD44 and TNFRSF18 exhibited positive correlations with PRMTScore, whereas other ICPs displayed inverse correlations (Figure 7D). To offer further insight into the TME, the ESTIMATE algorithm was applied to determine TME scores across PRMTGroups, revealing a higher immune score and a lower stromal score in the high PRMTGroup, suggestive of an enriched immune-related composition (Figure 7E). Notably, a heatmap was constructed to illustrate the distribution of tumor purity, TME scores, and the abundance of immune-related cell types across PRMTGroups (Figure 7F and Table S14).

Furthermore, we explored the immunotherapy response in ccRCC patient subgroups stratified by the PRMTScore. We first calculated the TIDE score of each patient. As shown in Figure 7G, patients in low PRMTGroup had a lower TIDE score, which implied that patients with the low PRMTScore may be more sensitive to immunotherapy. External validation using the David A. Braun and David Liu cohorts reaffirmed these trends, whereby patients situated in the low PRMTGroup exhibited a more favorable prognosis, aligning with heightened potential for benefiting from ICI therapy (Figure 7H-I). In conclusion, our findings suggested that the PRMTScore emerged as a potent predictor of the clinical response to immunotherapy in ccRCC patients.

### PRMT5 Plays an Anticancer Role in ccRCC

PRMT5 is the hottest research frontier in the field of protein arginine methylation 21. It's widely believed that PRMT5 is associated with oncogenic processes in many tumors, such as leukemia, glioblastoma, and prostate cancer 45,46,47. Moreover, several inhibitors targeting PRMT5 have entered clinical development for patients with hematological malignancies and advanced solid tumors 19. However, the role of PRMT5 in ccRCC remains enigmatic. In the above results, we found that PRMT5 was down-regulated in the transcriptome data of ccRCC (**Figure 1A**). Moreover, the ccRCC patient with high expression of PRMT5 had an excellent prognosis (**Figure S1F**). Therefore, we proposed a hypothesis that PRMT5 may play an anticancer function in ccRCC.

**Figure 8:**
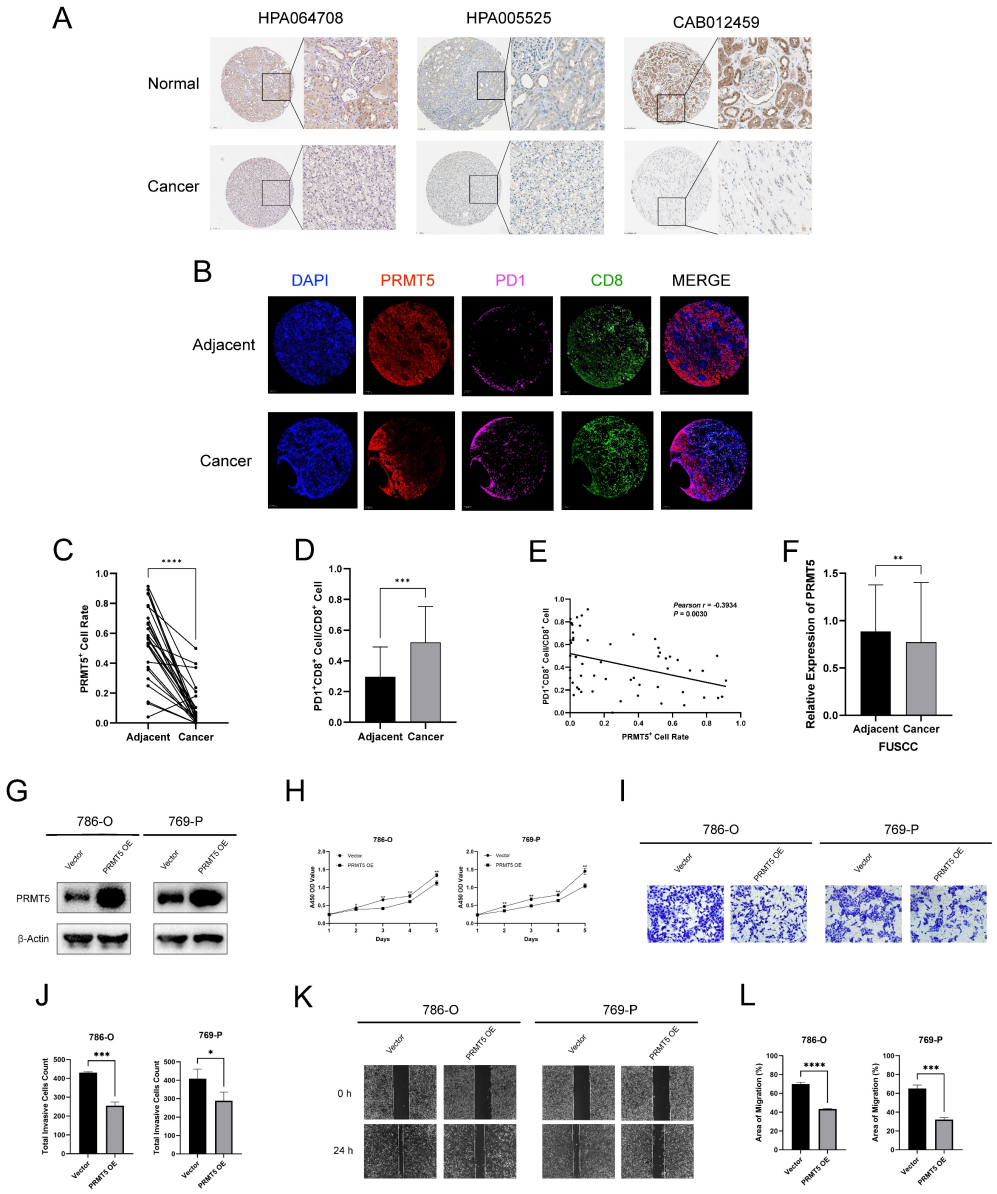
PRMT5 Plays an Anticancer Role in ccRCC. (A) Immunohistochemistry staining of PRMT5 protein expression from the HPA database. (B) Representative multiplex immunohistochemistry (mIHC) images show the positivity of PRMT5, PD1, and CD8 in 30 paired ccRCC and adjacent tissues. (C) The PRMT5^+^ cell rate of mIHC in 30 paired ccRCC and adjacent tissues. (D) The ratio of PD1^+^CD8^+^ cell and CD8^+^ cell of mIHC in 30 paired ccRCC and adjacent tissues. (E) The correlation between PRMT5^+^ cell rate and the abundance of PD1^+^CD8^+^ cell of mIHC in 30 paired ccRCC and adjacent tissues. (F) The relative protein expression of PRMT5 in 232 paired ccRCC and adjacent tissues from FUSCC cohorts. (G) Western blotting for PRMT5 protein expression level in 786-O and 769-P cells. (H) The CCK-8 assay of 786-O and 769-P cells transfected with vector or PRMT5 overexpression plasmid. (I-J) The transwell assay of 786-O and 769-P cells transfected with vector or PRMT5 overexpression plasmid. (K-L) The wound healing assay of 786-O and 769-P cells transfected with vector or PRMT5 overexpression plasmid. (ns, not significant; *p < 0.05, **p < 0.01, ***p < 0.001, ****p < 0.0001).

We first explored the IHC staining of PRMT5 protein from the HPA database, which confirmed that PRMT5 was down-regulated in renal cancers at the protein level **(Figure 8A)**. Thereafter, we applied mIHC to characterize the cellular composition of cancer and adjacent tissue for 30 ccRCC patients. We found that the expression of PRMT5 was significantly lower in cancer, compared with the adjacent normal tissues. (**Figure 8B and 8C**). Moreover, the abundance of PD1^+^ CD8^+^ cells was higher in cancer, implying that the immune microenvironment was relatively exhausted (**Figure 8B and 8D**). Moreover, the expression of PRMT5 was negatively correlated with the abundance of PD1^+^ CD8^+^ cells (**Figure 8B and 8E**). The FUSCC cohort comprising 232 ccRCC and adjacent samples also demonstrated that PRMT5 was low-expression in ccRCC at the protein level (**Figure 8F**).

Furthermore, we explored the biological role of PRMT5 in ccRCC *in vitro*. We transfected human ccRCC cells (786-O and 769-P) with PRMT5 overexpression plasmid, and western blotting was conducted to verify the transfection efficiency (**Figure 8G**). The CCK-8 assay showed that the proliferative ability of the cells with PRMT5 overexpression was significantly inhibited (**Figure 8H**). In transwell assay, the number of invasive cells decreased in the PRMT5 overexpression group (**Figure 8I-J**). Besides, the wound healing assay demonstrated that, in PRMT5 overexpression group, the migratory ability of the cells reduced (**Figure 8K-L**).

Taken together, the comprehensive investigation unveiled that PRMT5 was down-regulated in ccRCC at the mRNA and protein levels, and the low expression of PRMT5 was correlated with a malignant prognosis. Importantly, functional experiments suggested an anticancer role for PRMT5 in ccRCC, underscored by inhibiting the proliferative, invasive, and migratory abilities.

## Discussion

Despite remarkable advances in the diagnosis and management of ccRCC patients during the last two decades, ccRCC remains one of the most lethal urological malignancies 48. With the rapid development of cancer immunotherapy, treatment strategies targeting the immune suppression state in TME have contributed to dramatic clinical improvements for ccRCC patients. Immunotherapy-based combinatorial therapies are now transforming the treatment paradigm for patients with ccRCC 15. Nonetheless, a substantial proportion of ccRCC patients are resistant to the ICI therapy and do not derive persistent benefits 49. Therefore, it's an urgent need to explore new models for predicting prognostic outcomes and immunotherapy responses of ccRCC patients, guiding better individualized treatment strategies and facilitating enhancements in the clinical efficacy of immunotherapy.

The realm of protein arginine methylation, recognized as a pivotal epigenetic modification, exerts profound influence over diverse fundamental biological processes 19. Notably, growing evidence suggests that PRMTs are closely involved in cancer immunity, which has highlighted their emerging as attractive immunotherapeutic targets 24. However, in the field of ccRCC research, the precise impacts of PRMTs have not been well described, especially the subject of how PRMTs affect the immune characteristics of ccRCC 32.

To the best of our knowledge, this is the first study to construct an arginine methylation modification signature for the prediction of prognostic outcomes in cancer patients. In our research, based on the expressions of 8 PRMTs, we stratified ccRCC patients into two arginine methylation modification subgroups (PRMTCluster A and B) with the consensus clustering algorithm. The two subgroups showed significant differences in clinical outcomes, biological functions, and TME characteristics. Subsequently, we identified the DEGs between the subgroups and performed uniCox analysis to obtain the prognosis-related genes. Moreover, the consensus clustering algorithm was applied again to classify ccRCC patients into different genetic clusters (GeneCluster A-C) based on the 294 prognosis-related genes. Similar to the clustering results of the arginine methylation modification phenotypes, the three genetic subtypes also showed apparent differences. The aforementioned results revealed that arginine methylation modification contributed to the formation of complex TME, and thereby affected the prognostic outcomes of ccRCC patients.

Furthermore, to evaluate the role of arginine methylation modification in individual ccRCC patients, we established a scoring system (PRMTScore). The four most compelling prognosis-related genes (*SLC16A12*, *HRH2*, *F2RL3*, and *SAA1*) were identified to calculate the PRMTScore of individual patients by LASSO and multivariate Cox analysis. We found that ccRCC patients in PRMTCluster B and GeneCluster C had a higher PRMTScore, associated with a poor prognosis. We then conducted ROC curves to validate the predictive reliability of the PRMTScore system. The AUCs (0.709, 0.693, and 0.695, respectively) implied an excellent performance of the signature in predicting the prognosis for the 1-, 3-, and 5-year OS. Among the ccRCC patient subgroups stratified by different clinical factors (tumor grade, TNM stage, and TMB), the PRMTScore also showed stable predictive efficacy. Considering the robust predictive power of the PRMTScore, we constructed a nomogram that integrated the PRMTScore and clinical characteristics (age, gender, tumor grade, and TNM stage) to guide the clinical management of ccRCC patients. In addition, we explored the differences in TME characteristics, especially immune fractions, between the PRMTGroups. The high PRMTGroup had a higher immune score and lower stromal score, indicating that arginine methylation modification affected the abundance of immune and stromal fractions in ccRCC. We found that the abundance of 22 immune cell types and the expression levels of 26 ICPs showed significant differences between the two PRMTGroups. Moreover, we evaluated the ICI therapeutic response of ccRCC patients in the two PRMTGroups with the TIDE algorithm, indicating that patients with low PRMTScore benefited more from ICI therapy. The predictive result was well validated by two external ICI-treated cohorts. Taken together, the PRMTScore could be an ideal tool for predicting the prognostic outcomes, TME characteristics, and immunotherapy response in ccRCC patients.

In patients who receive ICI treatment, responders typically show a “hot” (“immune-inflamed”) phenotype, characterized by the presence of T lymphocytes in the tumor parenchyma, whereas non-responders usually exhibit a “cold” (“immune-excluded”/ “immune-desert”) phenotype, characterized by the exclusion or absence of T lymphocytes 50. However, increasing findings suggest that the infiltration level of T cells might be necessary but insufficient to define “hot” and “cold” tumors, which raised questions on how to identify ICI responders precisely 51. In this study, we identified that ccRCC patients in low PRMTGroup were responders to ICI treatment, and they exhibited a lower abundance of T cells regulatory (Tregs) and a higher level of macrophages M1. Tregs, an immunosuppressive T cells subset, were associated with weaker anticancer immune response and worse OS in cancer patients 52. Macrophages M1 within the TME can induce the initiation of inflammatory and immune responses, widely is widely considered to play a proinflammatory and anticancer role 53. Therefore, it is inappropriate to define “hot” tumors based only on the abundance of T cells; rather, different subtypes and ratios of T cells and other immune cells in the TME should be taken into consideration for the identification of the responders to ICI treatment.

PRMT5 is at the forefront in the research field of protein arginine methylation. It's commonly recognized that PRMT5 is an important oncogene, and several small-molecule inhibitors targeting PRMT5 have been identified for cancer patients 19. Only one research has reported on the role of PRMT5 in ccRCC, and it proposed that LINC01138 can interact with PRMT5, thereby promoting lipid desaturation and cell proliferation in ccRCC 54. However, the main focus of this study was the function of LINC01138 in ccRCC, and the authors only made a preliminary exploration of PRMT5. Therefore, more studies are required to elucidate the role of PRMT5 in ccRCC. In our current study, we found that PRMT5 was significantly down-regulated in ccRCC and related to a better prognosis for patients from TCGA, CPTAC, EMBL, and ICGC databases at transcription level. Thereafter, IHC staining of PRMT5 protein in renal cancers from the HPA database and proteomic data from the FUSCC cohort including 232 ccRCC patients confirmed the low-expression of PRMT5 in ccRCC at the protein level. Moreover, we discovered that the expression of PRMT5 was significantly lower in cancer and was negatively correlated with the abundance of PD1^+^ CD8^+^ cells in 30 ccRCC patients by mIHC. Besides, the proliferative, invasive, and migratoty abilities of human ccRCC cells were inhibited in the PRMT5 overexpression group. These results suggested that PRMT5 acted as a cancer suppressor in ccRCC.

However, several limitations of this study should be noted. First, the results were constructed and validated retrospectively by the information of ccRCC patients from public databases. To further validate the discoveries, more prospective studies are required. Second, demographic differences among the cohorts introduced an unavoidable bias in this study. Third, we did not deeply clarify the role and mechanism of individual PRMT in ccRCC; hence, more comprehensive experiments should be conducted *in vivo* and *in vitro* to gain further insight into the relationship between PRMTs and ccRCC.

## Conclusion

In conclusion, this study elucidated the underlying relationship between tumor epigenetic heterogeneity and immune characteristics. Our findings demonstrated that protein arginine methylation modification played a pivotal role in the formation of complex TME in ccRCC. We constructed a protein arginine methylation-related signature for predicting prognostic outcomes, TME characteristics, and immunotherapy response in ccRCC patients. This robust predictive tool may help clinicians in making more precise treatment decisions for ccRCC patients. Moreover, this was the first study to propose the anticancer role of PRMT5 in ccRCC.

## Supplementary Material

Supplementary figures.

Supplementary tables.

## Abbreviations

accRCC: advanced clear cell renal cell carcinoma
aDMA: ω‐*N^G^*,*N^G^*‐asymmetric dimethylarginine
ccRCC: clear cell renal cell carcinoma
CDF: cumulative distribution function
CNVs: copy number variations
CPTAC: Clinical Proteomic Tumor Analysis Consortium
CTL: cytotoxic T lymphocyte
DCA: decision curve analysis
DEGs: differential expression genes
EMBL: European Molecular Biology Laboratory
FDA: Food and Drug Administration
FUSCC: Fudan University Shanghai Cancer Center
GO: Gene Ontology
GSVA: gene set variation analysis
HPA: the Human Protein Altas
ICGC: International Cancer Genome Consortium
ICI: immune checkpoint inhibitor
ICPs: immune checkpoints
IHC: immunohistochemistry
KEGG: Kyoto Encyclopedia of Genes and Genomes
LASSO: least absolute shrinkage and selector operation
mIHC: multiplex immunohistochemistry
MMA: ω‐*N^G^*‐monomethylarginine
mTOR: mammalian target of rapamycin
NSCLC: non-small-cell lung cancer
OS: overall survival
PCA: principal component analysis
PPI: protein protein interaction
PRMTs: protein arginine methyltransferases
PTMs: post-translational modifications
RCC: renal cell carcinoma
ROC: receiver operating characteristic
SAM: S-adenosylmethionine
sDMA: ω‐*N^G^*,*N^'G^*‐symmetric dimethylarginine
ssGSEA: single-sample gene set enrichment analysis
TCGA: the Cancer Genome Atlas
TIDE: tumor immune dysfunction and exclusion
TMB: tumor mutation burden
TME: tumor microenvironment
VEGF: vascular endothelial growth factor

## References

[1] Siegel RL, Miller KD, Fuchs HE, Jemal A (2022). Cancer statistics, 2022. CA Cancer J Clin.

[2] Zheng RS, Zhang SW, Zeng HM, Wang SM, Sun KX, Chen R (2022). Cancer incidence and mortality in China, 2016. Journal of the National Cancer Center.

[3] Wei JH, Feng ZH, Cao Y, Zhao HW, Chen ZH, Liao B (2019). Predictive value of single-nucleotide polymorphism signature for recurrence in localised renal cell carcinoma: a retrospective analysis and multicentre validation study. Lancet Oncol.

[4] Wettersten HI, Aboud OA, Lara PN Jr, Weiss RH (2017). Metabolic reprogramming in clear cell renal cell carcinoma. Nat Rev Nephrol.

[5] Xu WH, Anwaier A, Liu WR, Tian X, Su JQ, Shi GH (2022). The unique genomic landscape and prognostic mutational signature of Chinese clear cell renal cell carcinoma. Journal of the National Cancer Center.

[6] Gerlinger M, Horswell S, Larkin J, Rowan AJ, Salm MP, Varela I (2014). Genomic architecture and evolution of clear cell renal cell carcinomas defined by multiregion sequencing. Nat Genet.

[7] Barata PC, Rini BI (2017). Treatment of renal cell carcinoma: Current status and future directions. CA Cancer J Clin.

[8] Hsieh JJ, Purdue MP, Signoretti S, Swanton C, Albiges L, Schmidinger M (2017). Renal cell carcinoma. Nat Rev Dis Primers.

[9] Garon EB, Rizvi NA, Hui R, Leighl N, Balmanoukian AS, Eder JP (2015). Pembrolizumab for the treatment of non-small-cell lung cancer. N Engl J Med.

[10] Larkin J, Hodi FS, Wolchok JD (2015). Combined Nivolumab and Ipilimumab or Monotherapy in Untreated Melanoma. N Engl J Med.

[11] Homet Moreno B, Ribas A (2015). Anti-programmed cell death protein-1/ligand-1 therapy in different cancers. Br J Cancer.

[12] Xu JX, Maher VE, Zhang L, Tang S, Sridhara R, Ibrahim A (2017). FDA Approval Summary: Nivolumab in Advanced Renal Cell Carcinoma After Anti-Angiogenic Therapy and Exploratory Predictive Biomarker Analysis. Oncologist.

[13] Bedke J, Albiges L, Capitanio U, Giles RH, Hora M, Lam TB (2021). The 2021 Updated European Association of Urology Guidelines on Renal Cell Carcinoma: Immune Checkpoint Inhibitor-based Combination Therapies for Treatment-naive Metastatic Clear-cell Renal Cell Carcinoma Are Standard of Care. Eur Urol.

[14] Rini BI, Battle D, Figlin RA, George DJ, Hammers H, Hutson T (2019). The society for immunotherapy of cancer consensus statement on immunotherapy for the treatment of advanced renal cell carcinoma (RCC). J Immunother Cancer.

[15] Díaz-Montero CM, Rini BI, Finke JH (2020). The immunology of renal cell carcinoma. Nat Rev Nephrol.

[16] Braun DA, Bakouny Z, Hirsch L, Flippot R, Van Allen EM, Wu CJ (2021). Beyond conventional immune-checkpoint inhibition - novel immunotherapies for renal cell carcinoma. Nat Rev Clin Oncol.

[17] Blanc RS, Richard S (2017). Arginine Methylation: The Coming of Age. Mol Cell.

[18] Yang Y, Bedford MT (2013). Protein arginine methyltransferases and cancer. Nat Rev Cancer.

[19] Wu Q, Schapira M, Arrowsmith CH, Barsyte-Lovejoy D (2021). Protein arginine methylation: from enigmatic functions to therapeutic targeting. Nat Rev Drug Discov.

[20] Wesche J, Kühn S, Kessler BM, Salton M, Wolf A (2017). Protein arginine methylation: a prominent modification and its demethylation. Cell Mol Life Sci.

[21] Xu J, Richard S (2021). Cellular pathways influenced by protein arginine methylation: Implications for cancer. Mol Cell.

[22] Guccione E, Richard S (2019). The regulation, functions and clinical relevance of arginine methylation. Nat Rev Mol Cell Biol.

[23] Jarrold J, Davies CC (2019). PRMTs and Arginine Methylation: Cancer's Best-Kept Secret?. Trends Mol Med.

[24] Dai W, Zhang J, Li S, He F, Liu Q, Gong J (2022). Protein Arginine Methylation: An Emerging Modification in Cancer Immunity and Immunotherapy. Front Immunol.

[25] Kim H, Kim H, Feng Y, Li Y, Tamiya H, Tocci S (2020). PRMT5 control of cGAS/STING and NLRC5 pathways defines melanoma response to antitumor immunity. Sci Transl Med.

[26] Hou J, Wang Y, Shi L, Chen Y, Xu C, Saeedi A (2021). Integrating genome-wide CRISPR immune screen with multi-omic clinical data reveals distinct classes of tumor intrinsic immune regulators. J Immunother Cancer.

[27] Zheng NN, Zhou M, Sun F, Huai MX, Zhang Y, Qu CY (2020). Combining protein arginine methyltransferase inhibitor and anti-programmed death-ligand-1 inhibits pancreatic cancer progression. World J Gastroenterol.

[28] Lu SX, De Neef E, Thomas JD, Sabio E, Rousseau B, Gigoux M (2021). Pharmacologic modulation of RNA splicing enhances anti-tumor immunity. Cell.

[29] Wang J, Wang C, Xu P, Li X, Lu Y, Jin D (2021). PRMT1 is a novel molecular therapeutic target for clear cell renal cell carcinoma. Theranostics.

[30] Liu K, Ma J, Ao J, Mu L, Wang Y, Qian Y (2021). The Oncogenic Role and Immune Infiltration for CARM1 Identified by Pancancer Analysis. J Oncol.

[31] Liu F, Wan L, Zou H, Pan Z, Zhou W, Lu X (2020). PRMT7 promotes the growth of renal cell carcinoma through modulating the β-catenin/C-MYC axis. Int J Biochem Cell Biol.

[32] Zhang C, Zhuang S (2020). The role of protein arginine methyltransferases in kidney diseases. Clin Sci (Lond).

[33] Wilkerson MD, Hayes DN (2010). ConsensusClusterPlus: a class discovery tool with confidence assessments and item tracking. Bioinformatics.

[34] Rich JT, Neely JG, Paniello RC, Voelker CC, Nussenbaum B, Wang EW (2010). A practical guide to understanding Kaplan-Meier curves. Otolaryngol Head Neck Surg.

[35] Chen B, Khodadoust MS, Liu CL, Newman AM, Alizadeh AA (2018). Profiling Tumor Infiltrating Immune Cells with CIBERSORT. Methods Mol Biol.

[36] Huang L, Wu C, Xu D, Cui Y, Tang J (2021). Screening of Important Factors in the Early Sepsis Stage Based on the Evaluation of ssGSEA Algorithm and ceRNA Regulatory Network. Evol Bioinform Online.

[37] Yoshihara K, Shahmoradgoli M, Martínez E, Vegesna R, Kim H, Torres-Garcia W (2013). Inferring tumour purity and stromal and immune cell admixture from expression data. Nat Commun.

[38] Ritchie ME, Phipson B, Wu D, Hu Y, Law CW, Shi W (2015). limma powers differential expression analyses for RNA-sequencing and microarray studies. Nucleic Acids Res.

[39] Yu G, Wang LG, Han Y, He QY (2012). clusterProfiler: an R package for comparing biological themes among gene clusters. OMICS.

[40] Hänzelmann S, Castelo R, Guinney J (2013). GSVA: gene set variation analysis for microarray and RNA-seq data. BMC Bioinformatics.

[41] Jiang P, Gu S, Pan D, Fu J, Sahu A, Hu X (2018). Signatures of T cell dysfunction and exclusion predict cancer immunotherapy response. Nat Med.

[42] Braun DA, Hou Y, Bakouny Z, Ficial M, Sant' Angelo M, Forman J (2020). Interplay of somatic alterations and immune infiltration modulates response to PD-1 blockade in advanced clear cell renal cell carcinoma. Nat Med.

[43] Liu D, Schilling B, Liu D, Sucker A, Livingstone E, Jerby-Arnon L (2019). Integrative molecular and clinical modeling of clinical outcomes to PD1 blockade in patients with metastatic melanoma. Nat Med.

[44] Mossmann D, Park S, Hall MN (2018). mTOR signaling and cellular metabolism are mutual determinants in cancer. Nat Rev Cancer.

[45] Greenblatt SM, Liu F, Nimer SD (2016). Arginine methyltransferases in normal and malignant hematopoiesis. Exp Hematol.

[46] Sachamitr P, Ho JC, Ciamponi FE, Alawi WB, Coutinho FJ, Guilhamon P (2021). PRMT5 inhibition disrupts splicing and stemness in glioblastoma. Nat Commun.

[47] Deng X, Shao G, Zhang HT, Li C, Zhang D, Cheng L (2017). Protein arginine methyltransferase 5 functions as an epigenetic activator of the androgen receptor to promote prostate cancer cell growth. Oncogene.

[48] Capitanio U, Bensalah K, Bex A, Boorjian SA, Bray F, Coleman J (2019). Epidemiology of Renal Cell Carcinoma. Eur Urol.

[49] Braun DA, Bakouny Z, Hirsch L, Flippot R, Van Allen EM, Wu CJ (2021). Beyond conventional immune-checkpoint inhibition - novel immunotherapies for renal cell carcinoma. Nat Rev Clin Oncol.

[50] Chen DS, Mellman I (2017). Elements of cancer immunity and the cancer-immune set point. Nature.

[51] Zhang J, Huang D, Saw PE, Song E (2022). Turning cold tumors hot: from molecular mechanisms to clinical applications. Trends Immunol.

[52] Knochelmann HM, Dwyer CJ, Bailey SR (2018). When worlds collide: Th17 and Treg cells in cancer and autoimmunity. Cell Mol Immunol.

[53] Xia Y, Rao L, Yao H, Wang Z, Ning P, Chen X (2020). Engineering Macrophages for Cancer Immunotherapy and Drug Delivery. Adv Mater.

[54] Zhang X, Wu J, Wu C, Chen W, Lin R, Zhou Y (2018). The LINC01138 interacts with PRMT5 to promote SREBP1-mediated lipid desaturation and cell growth in clear cell renal cell carcinoma. Biochem Biophys Res Commun.

