# Supplementary material for: Protein Arginine Methyltransferases Refine the Classification of Clear Cell Renal Cell Carcinoma with Distinct Prognosis and Tumor Microenvironment Characteristics
Source: Int J Biol Sci. 2023 Aug 28;19(14):4552–70. doi: 10.7150/ijbs.80323 (PMC10535715; doi:10.7150/ijbs.80323)
Supplement: Supplementary file 1 — Supplementary figures. [file ijbsv19p4552s1.pdf]

SUPPLEMENTARY FIGURE 1. Kaplan–Meier curves of the ccRCC patient subgroups stratified by the PRMTs expressions.
(A-H) Survival analyses of ccRCC patients subgroups stratified by the expressions of PRMT1, PRMT2, PRMT3, CARM1, PRMT5, PRMT6, PRMT7, and PRMT8.

SUPPLEMENTARY FIGURE 2. Identification of arginine methylation modification patterns based on the expression levels of the 8 PRMTs .
(A-H) Consensus matrices of ccRCC patients from k = 2 to k = 9.
(I-K) The cumulative distribution function (CDF) curves plot, delta plot, and tracking plot corresponding to the consensus matrices from k = 2 to k = 9.

SUPPLEMENTARY FIGURE 3. Generation of genetic clusters based on the DEGs and differential biological pathways among GeneClusters.
(A-H) Consensus matrices of ccRCC from k = 2 to k = 9.
(I-K) The CDF curves plot, delta plot, and tracking plot corresponding to the consensus matrices from k = 2 to k = 9.
(L-M) GSVA enrichment analysis showing the activation states of biological pathways among three GeneClusters.
(N) Abundance of immune cell types in different GeneClusters.
(O) Expressions of immune checkpoints (ICPs) in different GeneClusters.

SUPPLEMENTARY FIGURE 4. Correlation between clinical characteristics and the predictive signature.
(A-F) The proportions of clinical features (T stage, age, and gender) of ccRCC patients in different PRMTGroups.
(G-L) Kaplan–Meier analyses for patients in different PRMTGroups stratified by clinical features (T stage, age, and gender).

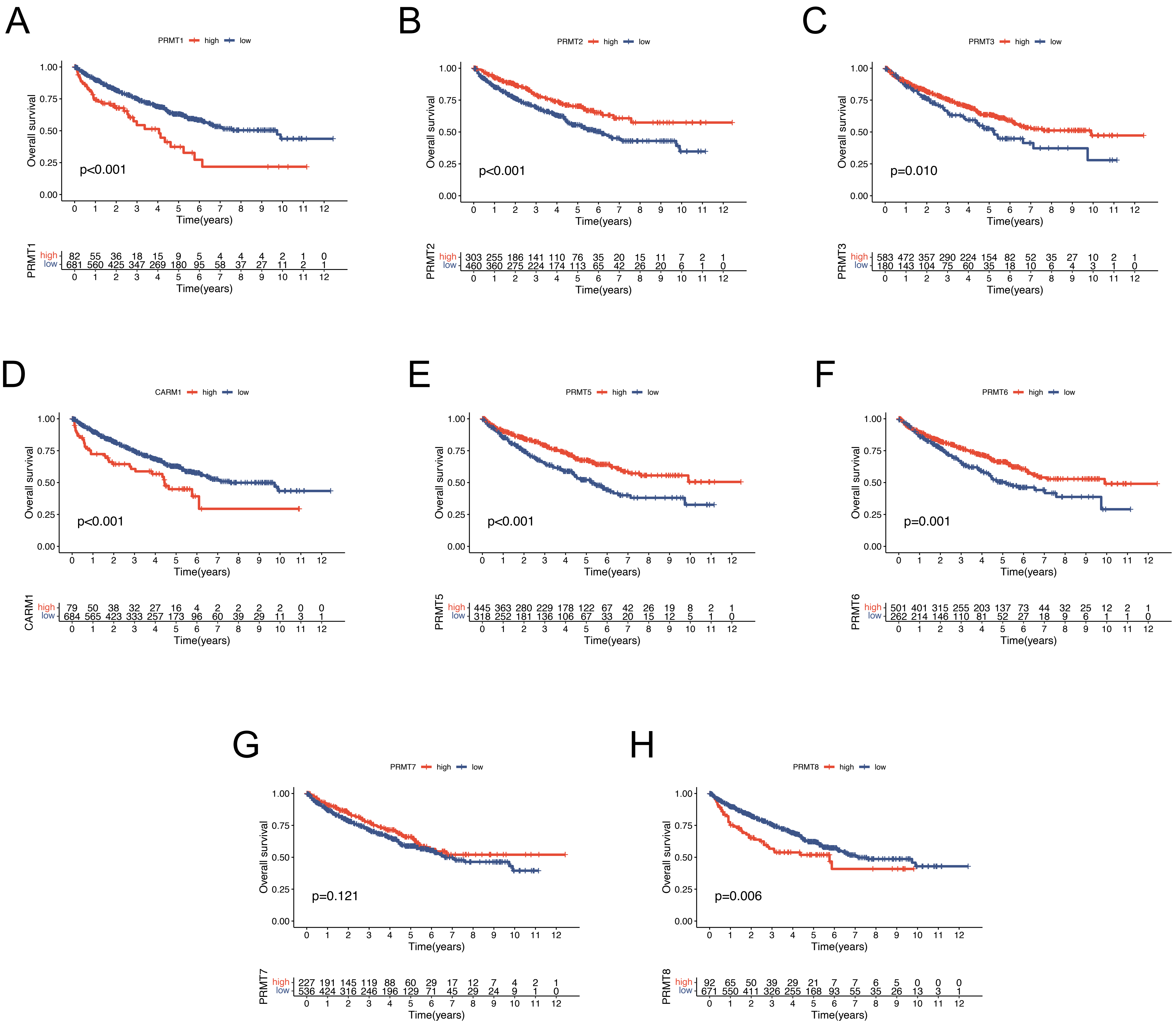
A
PRMT1 high low
p<0.001
B
PRMT2 high low
p<0.001
C
PRMT3 high low
p=0.010
D
CARM1 high low
p<0.001
E
PRMT5 high low
p<0.001
F
PRMT6 high low
p=0.001
G
PRMT7 high low
p=0.121
H
PRMT8 high low
p=0.006
Overall survival
Time(years)

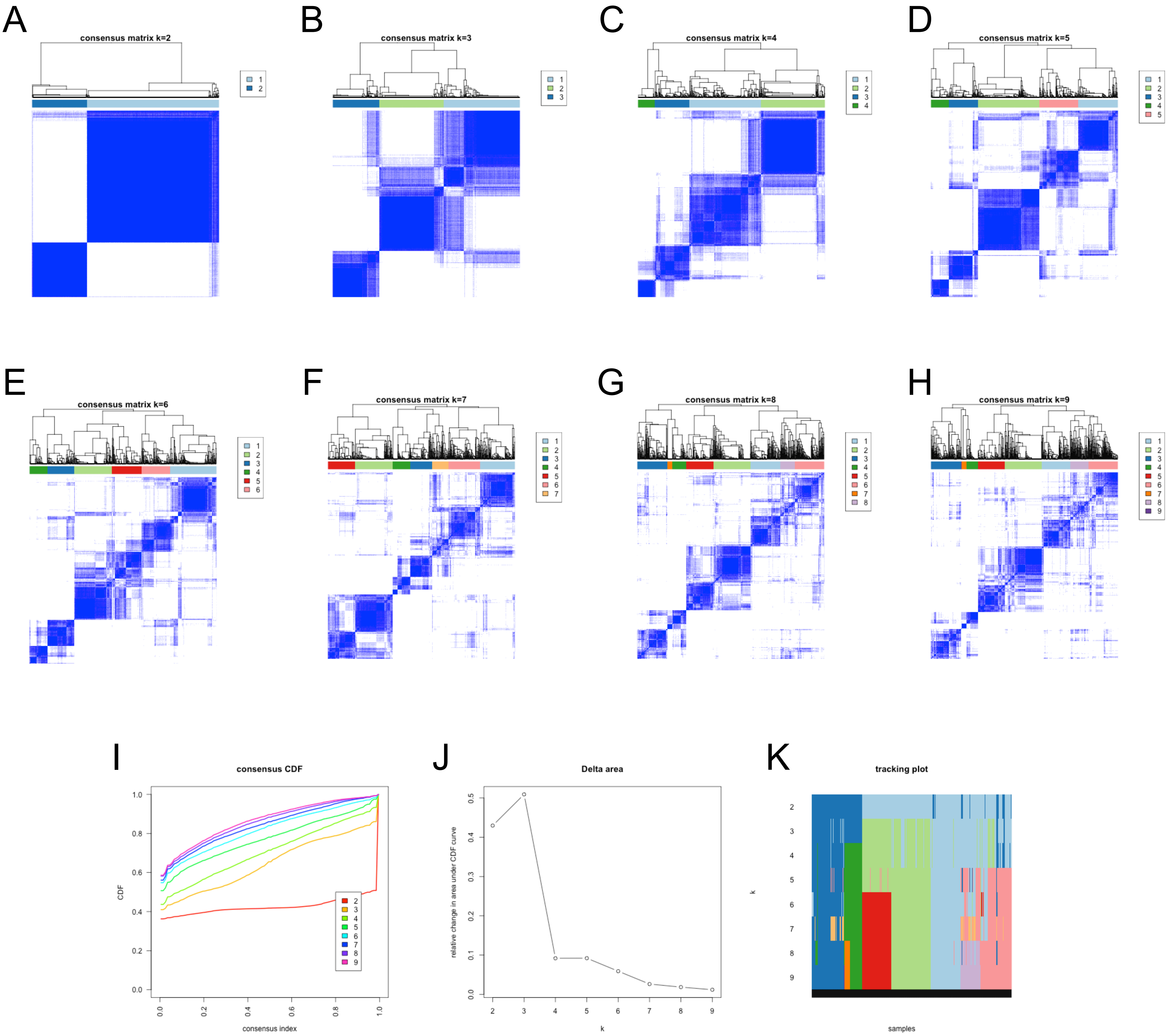
A
consensus matrix k=2
B
consensus matrix k=3
C
consensus matrix k=4
D
consensus matrix k=5
E
consensus matrix k=6
F
consensus matrix k=7
G
consensus matrix k=8
H
consensus matrix k=9
I
consensus CDF
CDF
consensus index
J
Delta area
relative change in area under CDF curve
k
K
tracking plot
k
samples

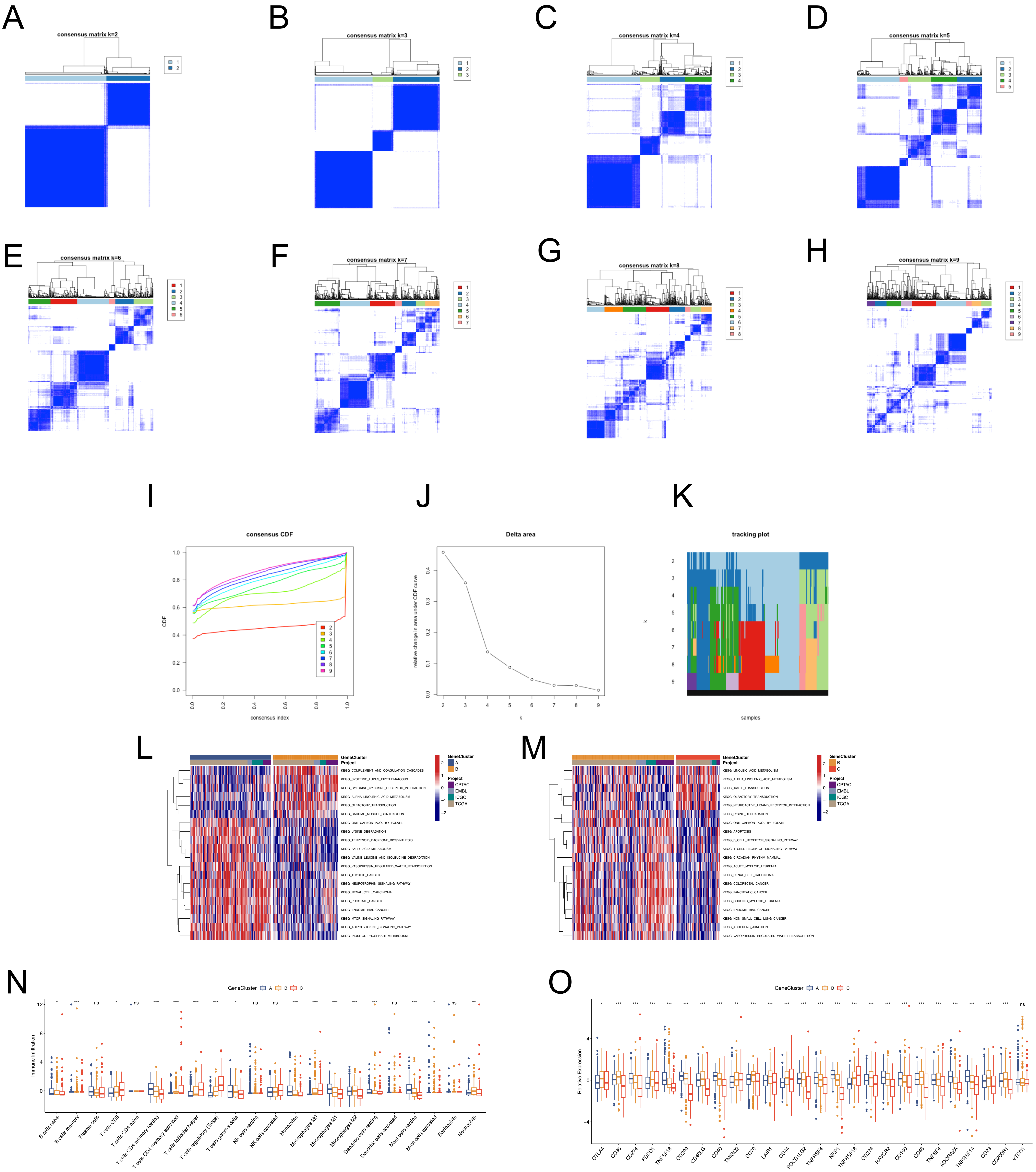
A
consensus matrix k=2
B
consensus matrix k=3
C
consensus matrix k=4
D
consensus matrix k=5
E
consensus matrix k=6
F
consensus matrix k=7
G
consensus matrix k=8
H
consensus matrix k=9
I
consensus CDF
CDF
consensus index
J
Delta area
relative change in area under CDF curve
k
K
tracking plot
k
samples
L
GeneCluster
Project
KEGG_COMPLEMENT_AND_COAGULATION_CASCADES
KEGG_SYSTEMIC_LUPUS_ERYTHEMATOSUS
KEGG_CYTOKINE_CYTOKINE_RECEPTOR_INTERACTION
KEGG_ALPHA_LINOLENIC_ACID_METABOLISM
KEGG_OLFACTORY_TRANSDUCTION
KEGG_CARDIAC_MUSCLE_CONTRACTION
KEGG_ONE_CARBON_POOL_BY_FOLATE
KEGG_LYSINE_DEGRADATION
KEGG_TERPENOID_BACKBONE_BIOSYNTHESIS
KEGG_FATTY_ACID_METABOLISM
KEGG_VALINE_LEUCINE_AND_ISOLEUCINE_DEGRADATION
KEGG_VASOPRESSIN_REGULATED_WATER_REABSORPTION
KEGG_THYROID_CANCER
KEGG_NEUROTROPHIN_SIGNALING_PATHWAY
KEGG_RENAL_CELL_CARCINOMA
KEGG_PROSTATE_CANCER
KEGG_ENDOMETRIAL_CANCER
KEGG_MTOR_SIGNALING_PATHWAY
KEGG_ADIPOCYTOKINE_SIGNALING_PATHWAY
KEGG_INOSITOL_PHOSPHATE_METABOLISM
GeneCluster
A
B
Project
CPTAC
EMBL
ICGC
TCGA
M
GeneCluster
Project
KEGG_LINOLEIC_ACID_METABOLISM
KEGG_ALPHA_LINOLENIC_ACID_METABOLISM
KEGG_TASTE_TRANSDUCTION
KEGG_OLFACTORY_TRANSDUCTION
KEGG_NEUROACTIVE_LIGAND_RECEPTOR_INTERACTION
KEGG_LYSINE_DEGRADATION
KEGG_ONE_CARBON_POOL_BY_FOLATE
KEGG_APOPTOSIS
KEGG_B_CELL_RECEPTOR_SIGNALING_PATHWAY
KEGG_T_CELL_RECEPTOR_SIGNALING_PATHWAY
KEGG_CIRCADIAN_RHYTHM_MAMMAL
KEGG_ACUTE_MYELOID_LEUKEMIA
KEGG_RENAL_CELL_CARCINOMA
KEGG_COLORECTAL_CANCER
KEGG_PANCREATIC_CANCER
KEGG_CHRONIC_MYELOID_LEUKEMIA
KEGG_ENDOMETRIAL_CANCER
KEGG_NON_SMALL_CELL_LUNG_CANCER
KEGG_ADHERENS_JUNCTION
KEGG_VASOPRESSIN_REGULATED_WATER_REABSORPTION
GeneCluster
B
C
Project
CPTAC
EMBL
ICGC
TCGA
N
GeneCluster A B C
Immune Infiltration
B cells naive
B cells memory
Plasma cells
T cells CD8
T cells CD4 naive
T cells CD4 memory resting
T cells CD4 memory activated
T cells follicular helper
T cells regulatory (Tregs)
T cells gamma delta
NK cells resting
NK cells activated
Monocytes
Macrophages M0
Macrophages M1
Macrophages M2
Dendritic cells resting
Dendritic cells activated
Mast cells resting
Mast cells activated
Eosinophils
Neutrophils
O
GeneCluster A B C
Relative Expression
CTLA4
CD86
CD274
PDCD1
TNFSF18
CD200
CD40LG
CD40
TMIGD2
CD70
LAIR1
CD44
PDCD1LG2
TNFRSF4
NRP1
TNFRSF18
CD276
HAVCR2
CD160
CD48
TNFSF4
ADORA2A
TNFRSF14
CD28
CD200R1
VTCN1

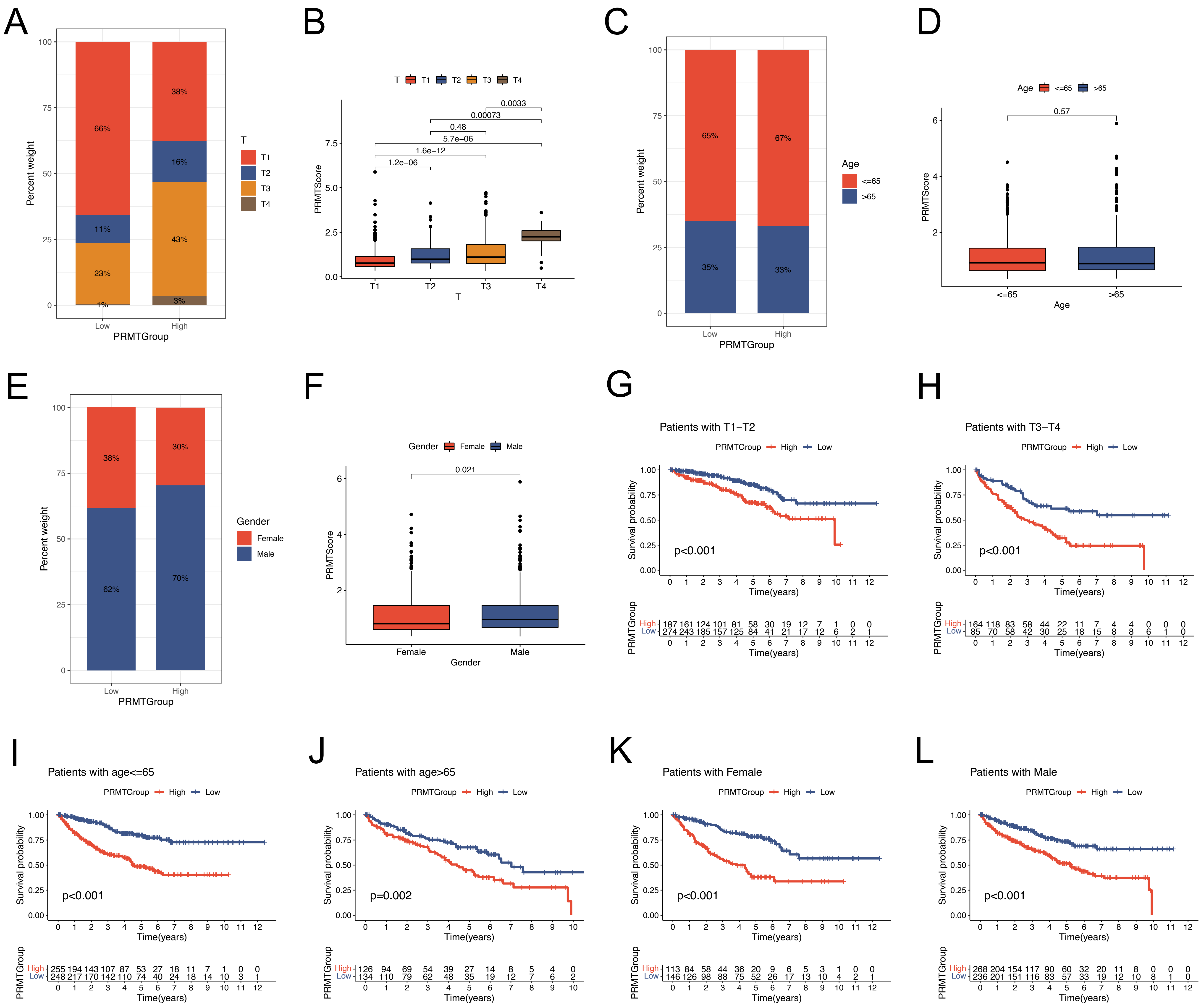
A
B
C
D
E
F
G
H
I
J
K
L
PRMTGroup
Percent weight
PRMTScore
Survival probability
Time(years)
Patients with T1−T2
Patients with T3−T4
Patients with age<=65
Patients with age>65
Patients with Female
Patients with Male
p<0.001
p=0.002
Gender
Female
Male
Age
0.021
0.57
1.2e−06
1.6e−12
5.7e−06
0.48
0.00073
0.0033
